# Role of Surface Hydroxyls in Atomic-Scale Copper Restructuring during CO Electroreduction

**DOI:** 10.1021/jacs.5c14516

**Published:** 2025-11-24

**Authors:** Jie Wei, Zisheng Zhang, Winston Gee, Yu Wei, Ya-Wei Zhou, Matias Herran, Philippe Sautet, Anastassia N. Alexandrova, Beatriz Roldan Cuenya, Christopher S. Kley

**Affiliations:** † Helmholtz Young Investigator Group Nanoscale Operando CO_2_ Photo-Electrocatalysis, 28340 Helmholtz-Zentrum Berlin für Materialien und Energie GmbH, 14109 Berlin, Germany; ‡ Department of Interface Science, Fritz Haber Institute of the Max Planck Society, 14195 Berlin, Germany; § Department of Chemistry and Biochemistry, 8783 University of California, Los Angeles, California 90095, United States; ∥ Department of Chemical and Biomolecular Engineering, University of California, Los Angeles, California 90095, United States; ⊥ SUNCAT Center for Interface Science and Catalysis, Department of Chemical Engineering, 6429 Stanford University, Stanford, California 94305, United States

## Abstract

The nanoscale structure of electrocatalyst surfaces governs the selectivity and kinetics of reactions including CO_(2)_ electroreduction (CO_(2)_R). Yet, their evolution under reaction conditions remains elusive, and the roles of surface hydroxyls (OH_ad_) and the interfacial microenvironment in surface restructuring are poorly understood. Combining electrochemical atomic force microscopy, Raman spectroscopy, and grand canonical modeling, we reveal that OH_ad_ acts synergistically with CO_ad_ to restructure copper (Cu) electrocatalysts during COR. Mixed OH_ad_/CO_ad_ coverage promotes lifting of surface atoms into metastable states, generating Cu adatoms and nanoclusters at mild cathodic potentials, which aggregate or dissolve at more negative potentials. This restructuring into low-coordinated Cu sites is accompanied by disordering of the interfacial water network. Nanocluster stability depends critically on CO partial pressure, while hydroxyls remain kinetically trapped on the roughened Cu surface. These findings underscore the importance of surface kinetics and interfacial microenvironments in atomic-scale surface restructuring, urging a reassessment of catalytic surface states under realistic conditions.

## Graphical abstract

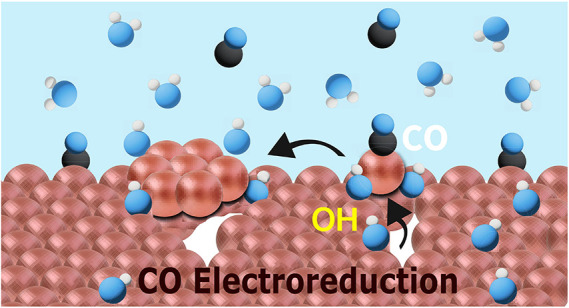

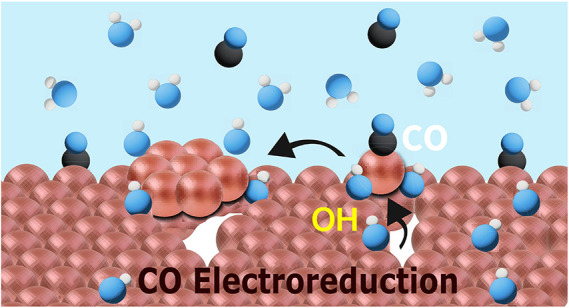

## Introduction

As energy conversion and storage technologies become essential to addressing the intermittency of renewable energy sources and mitigating anthropogenic carbon emissions, electrocatalysis has garnered significant attention.^1–3^ However, the economic feasibility of key electrocatalytic reactions, such as CO_(2)_ electroreduction (CO_(2)_R), hydrogen evolution (HER), and oxygen evolution (OER), remains constrained by moderate product selectivity and yields, energy efficiency, and performance stability. These factors are strongly influenced by the structure of the electrode surfaces where catalysis occurs.^4–8^ Thus, improving a catalyst’s selectivity and stability requires fundamental understanding of electrode surface structures while at work, and their modulation by factors such as surface/interfacial species and applied potential. Despite extensive research, catalyst surfaces during electrocatalytic reactions remain largely unresolved due to the challenge of probing electrochemical interfaces under relevant gas-evolving reaction conditions using in situ microscopy^9,10^ At the same time, realistic theoretical modeling of these interfaces requires innovative approaches to not only resolve an electrode’s thermodynamically preferred state, but also identify kinetically trapped metastable states. Moreover, the complex interplay between surface adsorbates, ionic species, and interfacial hydration layers complicates the identification of the factors governing the structure of a polarized electrode, and consequently, the electrocatalytic performance.^11–13^ These central limitations in electrocatalysis impede developing strategies for the rational design and ultimately *operando* control of catalysts.

Particularly, copper electrocatalysts undergo significant restructuring during CO_(2)_R, a promising route for converting atmospheric CO_(2)_ into valuable carbon-based fuels and chemicals.^14–18^ Recent studies suggest that nanoscale restructuring of Cu surfaces is related to interactions with CO,^19–21^ soluble intermediates,^22,23^ or Cu oxide reduction.^24,25^ While Cu catalyst restructuring directly influences specific CO_(2)_R pathways, the underlying mechanisms and the behavior of reconstructed Cu sites at relevant potentials and chemical environments remain elusive. In particular, the role of adsorbates beyond CO in surface restructuring is underexplored. In this context, surface hydroxyls (OH_ad_), alongside CO, play a key role in CO_(2)_R. Local alkaline microenvironments and coadsorbed OH have been shown to impact CO_(2)_R activity and selectivity by suppressing hydrogen evolution, altering CO adsorption energies, and the rate of C–C coupling.^26–33^ Therefore, disentangling the structural impact of OH_ad_ from CO_ad_ on catalyst surfaces is critical to understand the contributions of each coadsorbed surface species. While OH and CO coadsorption have been found to induce surface restructuring at the onset of CO oxidation on Cu(111),^34^ the influence of OH_ad_ on surface restructuring under cathodic potentials is still poorly understood. Recent studies suggest that coadsorbed OH promotes the formation of oxidative Cu species, irreversible changes in Cu morphology, and faceting.^15,26,35^ The explicit link between the local CO/OH_ad_ balance and the structural evolution of the catalyst surface remains experimentally unresolved. Addressing this gap requires a quantitative, mechanistic understanding of the surface states and the factors driving nanoscale Cu restructuring during CO_2_RR, underscoring the need for in situ nanoscale characterization under relevant reaction conditions and varied chemical environments.

In this work, we elucidate the atomic-scale surface restructuring of single-crystalline Cu(100) electrodes during COR, resolving the interplay between key adsorbates (CO, OH) and dynamic surface restructuring. By combining in situ electrochemical atomic force microscopy (EC-AFM), shell-isolated nanoparticle-enhanced Raman spectroscopy (SHINERS), and grand-canonical modeling, we demonstrate that (sub)­monolayer low-coordinated Cu clusters form via elevation of CuCO­(OH)_2_ species from the atomically smooth surface under mixed CO_ad_ and OH_ad_ coverage at mild cathodic potentials. This reveals the important role of coadsorbed OH in driving the initiation and controlling the extent of Cu surface restructuring synergistically with CO, accompanied by disordering of the interfacial water structure. The release of Cu surface atoms and the trapping of OH_ad_ on the irreversibly reconstructed surface show kinetic effects under thermodynamically unstable conditions. This provides new insights into the in situ surface states of Cu electrocatalysts under relevant reaction conditions, addressing long-standing uncertainties about adsorbate-induced morphological catalyst evolution.

## Results and Discussion

To investigate Cu surface restructuring mechanisms, we study single-crystalline Cu electrodes under varying chemical environments using in situ EC-AFM, as illustrated in Figure 1a. The Cu(100) electrode was prepared following established protocols and exposed to the electrolyte under potential control to preserve its well-defined surface structure and prevent oxidation–reduction-induced reconstruction (see Methods).^19^ The cleanliness of the as-prepared Cu(100) surface was verified by cyclic voltammetry (CV) in Ar-sat. 0.1 M KOH (Figure S1a).^14,36^ Upon saturating the electrolyte with CO, the CV exhibits a distinct anodic current above ∼0 V vs the reversible hydrogen electrode (V_RHE_) and a cathodic current below ∼−0.2 V_RHE_ (Figure S1a), corresponding to CO oxidation and reduction, respectively. High-resolution EC-AFM images (Figure S1b) confirmed the atomically flat morphology of Cu(100) in Ar-sat. 0.1 M KOH at −0.15 V_RHE_, a non-COR potential near the potential of zero charge (PZC) for Cu(100). Notably, switching the electrolyte in situ from Ar- to CO-saturated electrolyte causes rapid roughening of the Cu(100) surface (Figure 1b). The initially atomically flat terraces with monatomic steps become covered by a layer of small nanoclusters. Line profiles (Figure S1c) show increased terrace height variations of 50–80 pm. Closer examination of the marked area in Figure 1b highlights that step edges of the two ∼20 nm sized Cu islands, formed previously through redeposition upon exposing to electrolyte, transform into curved islands, along with pronounced ad-nanostructures on the terraces (Figure S1d–h), indicating a dynamic rearrangement of the surface Cu atoms upon introducing CO.

**Figure 1:**
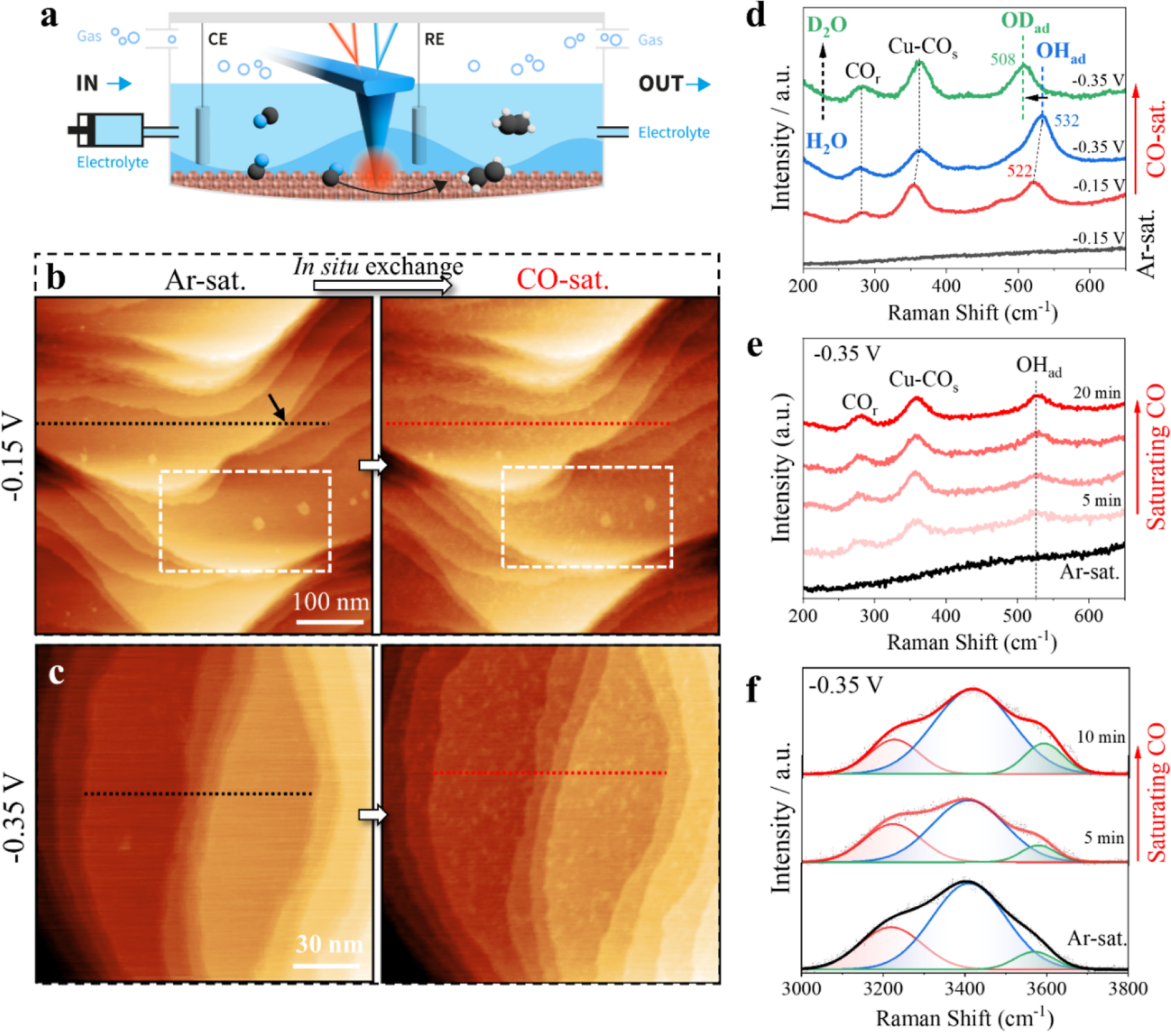
Nanoscale surface restructuring of Cu electrodes under CO and OH coadsorption. (a) Schematic of the electrochemical AFM (EC-AFM) cell, the in situ electrolyte exchange, and headspace gas atmosphere control. (b,c) Sequence of in situ EC-AFM images of Cu(100) recorded at −0.15 V_RHE_ (b) and −0.35 V_RHE_ (c) with the 0.1 M KOH electrolyte switched in situ from Ar- to CO-saturation. The images are taken 6 min after electrolyte exchange. (d–f) Normalized in situ SHINERS spectra recorded on the Cu(100) surface at −0.15 V_RHE_ and −0.35 V_RHE_ in Ar-sat. and CO-sat. 0.1 M KOH/KOD solutions.

High-resolution EC-AFM images further reveal two distinct lattice structures coexisting on the surface in CO-sat. 0.1 M KOH (Figure S1j). The smaller lattice corresponds to the bare Cu surface termination (Figure S1b), while the larger lattice matches the size and orientation of a Cu(100)-c(2 × 2)-CO or Cu(100)-(√2×√2) R45°-CO adlayer structure,^37^ as displayed in the top-view EC-AFM image. Over time, a disordered nanostructure (Figure S1k) evolves through atomic-scale roughening of Cu(100). Shifting the potential to −0.35 V_RHE_, the onset of COR, leads to further surface roughening and increased amounts of nanoclusters, with the round step edges of Cu islands becoming straightened. Switching the electrolyte in situ from Ar- to CO-saturation at −0.35 V_RHE_ led to a more pronounced formation of Cu nanoclusters on the previously smooth terrace (Figure 1c). Height variations on the Cu terrace reached about one monolayer (Figure S1l), exceeding those observed at −0.15 V_RHE_ (Figure S1c). This highlights the critical role of the potential-dependent interfacial microenvironment in forming low-coordinated Cu sites.

To gain molecular-level insights into how changes in the interfacial microenvironment influence Cu surface restructuring, we performed SHINERS measurements on Cu(100) in Ar- and CO-sat. 0.1 M KOH (Figure 1d). In CO-sat. electrolyte, two Raman bands at ∼276 cm^–1^ and 355 cm^–1^, assigned to CO rotation (CO_r_) and Cu–CO stretching (Cu–CO_s_) vibrations, respectively, appear alongside a new band centered at ∼522 cm^–1^ at −0.15 V_RHE_. As the potential decreases to −0.35 V_RHE_, the Cu–CO_s_ band slightly decreases, reflecting ongoing CO reduction, while the band at ∼522 cm^–1^ intensifies and shifts to ∼532 cm^–1^. This shift corresponds to a chemisorbed species subject to the vibrational Stark effect, with a Stark tuning rate of ∼50 cm^–1^/V, similar to that of the Cu–CO_s_ vibration. This vibrational fingerprint at ∼522 cm^–1^ aligns with the widely accepted assignment to the bending mode of chemisorbed surface hydroxyls (Cu–OH_ad_).^26–28,38–40^ Moreover, a red shift from ∼532 cm^–1^ to ∼508 cm^–1^ was observed in experiments using deuterated water (D_2_O) (Figures 1d and S2a,b). To rule out contributions from carbon species, we also conducted D_2_O and H_2_O^18^ labeling SHINERS experiments in Ar-saturated 0.1 M CsOH. The results reveal the same peak at ∼525 cm^–1^ as observed in CO-saturated 0.1 M KOH solution (Figure S2c), confirming its assignment to hydroxyl species adsorbed on Cu. This peak is associated with water dissociation, consistent with previous reports,^38^ and suggests kinetic effects for OH adsorption at cathodic potentials. The absence of the OH_ad_ band in Ar-sat. 0.1 M KOH under this potential regime suggests that CO coadsorption on Cu(100) at mild cathodic overpotentials enhances surface OH adsorption, which was previously linked to electronic effects or impact on the electric double layer (EDL) structure.^34,35,38,41,42^

Switching in situ from Ar- to CO-sat. 0.1 M KOH shows that the OH_ad_ signal appears simultaneously with the CO signal and intensifies with increasing CO saturation over time (Figure 1e). The O–H stretching region of water (ν­(O–H), 3000–3800 cm^–1^) can be deconvoluted into three Gaussian components, free or weakly hydrogen-bonded H_2_O (green), moderately hydrogen-bonded H_2_O (blue), and strongly hydrogen-bonded H_2_O (red) (Figure 1f).^43–46^ Upon CO saturation, the total O–H intensity decreases within 5 min, followed by a weakening of the hydrogen-bonding network as the fraction of weakly hydrogen-bonded H_2_O increases (Figures 1f and S2d). At −0.15 V_RHE_ in CO-sat. electrolyte, a higher proportion of free or weakly hydrogen-bonded H_2_O is observed (Figure S2e), indicating a disordered interfacial H_2_O structure upon CO saturation. These results suggest that CO promotes strong coadsorption with OH by tuning its adsorption energy and enhancing H_2_O dissociation,^47–49^ thereby extending the cathodic potential range over which surface *OH persists,^50^ critical for accurate interpretation of in situ surface states.

Inherently, the enhancement of surface OH adsorption must be critically considered in surface roughening and Cu nanocluster formation when transitioning from Ar- to CO-sat. 0.1 M KOH at these potentials. Direct exposure of Cu(100) to CO-sat. 0.1 M KOH yields terraces densely covered with pronounced Cu nanoclusters, in contrast to the smooth terraces observed in Ar-sat. electrolyte at both −0.15 and −0.35 V_RHE_ (Figure S3). Height distribution analysis identifies these features as submonolayer nanoclusters accompanied by shallow depressions (Figure S3e,f; marked area in Figure S3d), indicating that Cu surface vacancies form as terrace atoms migrate to assemble clusters. These results suggest that initial surface roughening and the formation of small nanoclusters commence at a relatively mild potential of −0.15 V_RHE_, preceding the onset of significant electrochemical CO consumption (−0.35 V_RHE_). The higher nanocluster density at −0.35 V_RHE_ correlates with increased OH_ad_/CO_ad_ coverage, suggesting that a mixed adlayer is required for this Cu surface restructuring into mono/submonolayer protrusions.

In situ EC-AFM in CO -sat. 0.05 M K_2_SO_4_ (pH ≈ 7), 0.1 M KHCO_3_ (pH ≈ 8.9), and 0.1 M H_2_SO_4_ (pH ≈ 1) show a distinct extent of surface restructuring at −0.35 V_RHE_: pronounced clusters in K_2_SO_4_ (Figure S4a), smoother terraces in KHCO_3_ (Figure S4b), and minimal restructuring in H_2_SO_4_ (Figure S4c). The solubility of CO in aqueous solution is low, and its concentration is not expected to vary significantly across the four electrolytes. SHINERS results confirm relatively strong CO adsorption in all cases, whereas markedly weaker OH_ad_ signals were detected in KHCO_3_ and H_2_SO_4_ solutions, likely due to the inhibition of local OH^–^ accumulation by HCO_3_^–^ buffering and high H^+^ concentration, respectively. This correlates well with the reduced surface restructuring observed in KHCO_3_ and H_2_SO_4_ solutions, suggesting that the local pH environment, rather than bulk pH, dictates the restructuring behavior. The specific identity of cations (K^+^ in this study) has been well-known to influence OH_ad_ stabilization through noncovalent interactions,^51,52^ which may also contribute to the low OH concentration in H_2_SO_4_ solution. Enhanced local alkalinity facilitates surface hydroxyl adsorption at cathodic potentials, which has been associated with promoting C_2_^+^ product formation.^26–33^ These findings collectively establish local alkalinity and surface OH_ad_ as key drivers of nanoscale Cu surface restructuring at mild cathodic potentials.

The formation of low-coordinated Cu sites at the onset of COR is critical for specific CO_(2)_R reaction pathways, yet their stability across the full COR potential range remains elusive. Previous studies have indicated undercoordinated Cu atoms throughout the CO_2_R potential range in bicarbonate electrolyte,^19^ while noting a tendency toward larger agglomerates at more negative potentials.^20^ To address the lack of in situ surface structure information at COR-relevant potentials, we next investigate the behaviors of these Cu nanoclusters at more cathodic potentials. Starting from a surface densely covered with Cu nanoclusters and vacancies at the onset of COR (−0.35 V_RHE_), in situ EC-AFM reveals that lowering the potential to −0.5 V_RHE_ produces larger vacancies and partial smoothing of Cu nanoclusters (Figure 2a,b, indicated by the red and yellow arrows, respectively). At −0.75 V_RHE_, terraces become smoother, Cu nanoclusters largely disappear, and the density of large vacancies on the surface increases (Figure 2c,d). Line profiles confirm the ripening of low-coordinated nanoclusters and vacancy growth at more cathodic potentials (Figure 2i). While the surface remains irreversibly roughened at the molecular scale, featuring Cu adatoms and vacancy defects on the terraces compared to atomically smooth terraces in Ar-sat. electrolyte (Figures 1b,c and S3a,c), this defect-rich state is more susceptible to restructuring, as evidenced by renewed progressive cluster formation when the potential is shifted back to −0.35 V_RHE_ (Figure S5).

**Figure 2:**
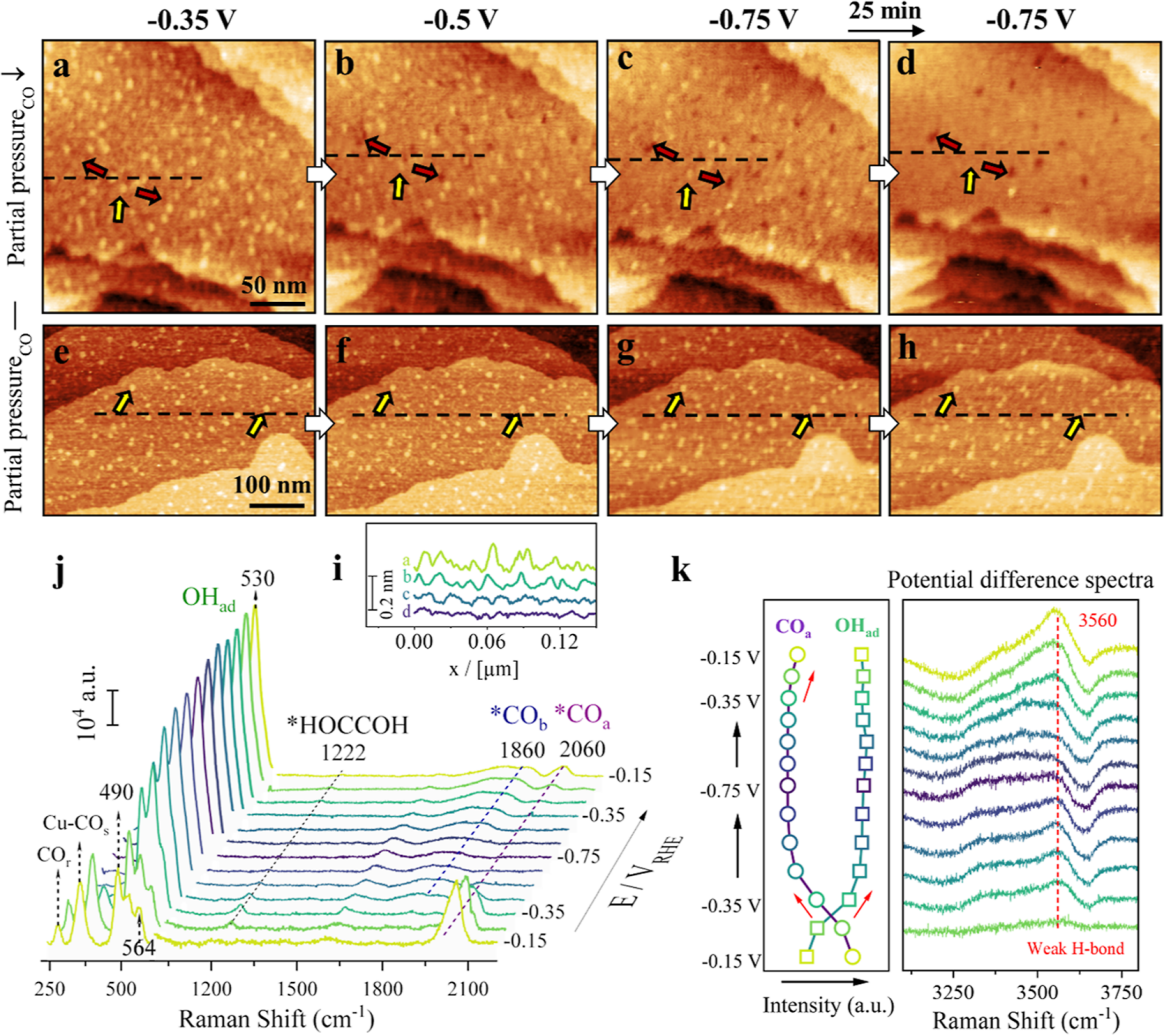
Nano- and molecular scale insights into Cu surfaces during CO electroreduction. (a–h) In situ EC-AFM images of as-prepared Cu(100) in CO-sat. 0.1 M KOH at different cathodic potentials, showing nanocluster dissolution at high cathodic potentials (a–d), while maintaining a constant CO partial pressure in the AFM chamber inhibits dissolution (e–h). (i) Height profiles along the horizontal lines in (a–d). (j) Potential-dependent in situ SHINER spectra of Cu(100) in CO-sat. 0.1 M KOH. (k) Potential-dependent intensities of OH_ad_, CO_ad_, and the difference spectra of the O–H stretching mode in CO-sat. 0.1 M KOH.

Zoomed-in EC-AFM images further highlight nanocluster annihilation at highly cathodic potentials and more pronounced surface restructuring of the previously roughened surface upon returning to mild cathodic potentials (Figure S6). Below −0.75 V_RHE_, Cu adatoms and nanoclusters aggregate into monatomic Cu islands (Figure S7), further indicating the instability of low-coordinated Cu species at highly cathodic potentials. Reintroducing CO gas into the AFM chamber headspace (as illustrated in Figure 1a) triggers a second restructuring cycle with higher nanocluster surface density (Figure S8). This suggests that the instability of low-coordinated Cu nanoclusters stems from CO depletion in the electrolyte. To confirm this, we examined the in situ Cu surface structure in a closed AFM chamber with a stable CO supply (∼0.1 mbar) through the headspace (Figure 1a). Under these conditions, Cu adatoms and nanoclusters remain highly stable even at −0.75 V_RHE_ (Figures 2e–h and S9a, highlighted by the yellow arrows), underscoring the critical role of stable CO partial pressure in inhibiting the ripening of low-coordinated nanoclusters across the COR potential regime.

Molecular-level insights into the Cu(100) surface chemical states across the entire COR potential range were obtained from potential-dependent in situ SHINER spectra in CO-sat. 0.1 M KOH (Figure 2j). *CO_a_, identified by its C≡O stretching band at ∼2060 cm^–1^ at −0.15 V_RHE_, corresponds to atop adsorption on terrace sites.^53–56^ As the potential shifts negatively, the *CO_a_ signal decreases due to rapid reduction to hydrocarbons, consistent with the emergence of a ∼1222 cm^–1^ band from −0.25 V_RHE_, assigned to long-lived *HOCCOH species formed via CO–CO coupling and reaction with surface water.^40^ The *HOCCOH band intensity peaks at −0.35 V_RHE_, and disappears along with *CO_a_ below −0.55 V_RHE_, likely due to rapid consumption of *HOCCOH under such conditions.^57^ Concurrently, a broad band at ∼2060 cm^–1^ emerges, corresponding to bridge-bound C=O (*CO_b_). This feature persists throughout both the cathodic and subsequent anodic potential shifts and is attributed to enhanced 2π* back-donation between the adsorbed *CO and metal surfaces at negative potentials–a configuration considered inactive toward further hydrocarbon formation.^58,59^ Notably, upon returning to −0.15 V_RHE_, the *CO_a_ signal remains significantly attenuated, indicating irreversible loss of pristine terrace sites.

A distinct triple peak for OH_ad_ bending modes is also resolved at 485–568 cm^–1^ when starting at −0.15 V_RHE_ in CO-sat. 0.1 M KOH due to strong CO coadsorption on Cu under this condition.^56,60^ This feature has been similarly reported for grain-boundary-rich Cu surfaces exhibiting weak *OH adsorption.^27,61^ The shoulder peak at ∼564 cm^–1^ is assigned to the libration mode of interfacial H_2_O,^44^ indicative of an ordered interfacial H_2_O structure. The OH_ad_ bending mode centered at ∼ 530 cm^–1^ becomes dominant from −0.35 V_RHE_ during fast CO reduction. This feature has also been attributed to strong OH adsorption on low-coordination amorphous Cu defect sites,^27,61^ which align with our EC-AFM results showing terraces densely covered with nanoclusters and vacancies at −0.35 V_RHE_ (Figures 2a,e and S3d). The disappearance of the ∼564 cm^–1^ shoulder peak at this potential suggests disruption of the ordered interfacial water network compared to the pristine Cu(100) surface prior to restructuring. The intense ∼530 cm^–1^ OH_ad_ bend continues to intensify at high overpotentials during rapid CO reduction, stabilizing below −0.55 V_RHE_ and persisting through the potential reversal to −0.15 V_RHE_. As shown in Figure 2k (left), while *CO_a_ is irreversibly lost after cathodic and anodic potential shifts, the OH_ad_ signal remains stable and pronounced after cathodic polarization. We note that such an OH_ad_ signal is absent in Ar-sat. 0.1 M KOH down to −0.55 V_RHE_ and during potential reversal (Figure S9b), confirming its independence from bulk OH^–^ concentration.

In addition, the interfacial H_2_O structure undergoes significant disordering as OH_ad_ coverage increases and *CO_a_ decreases, particularly under mixed CO and OH coverage at −0.35 V_RHE_ (Figure 2k). This is evident in the potential-difference spectra of the O–H stretching mode (Figure 2k, right panel) and is consistent with the disappearance of the ∼564 cm^–1^ libration mode of interfacial water. Using the spectrum at −0.15 V_RHE_ as baseline (original spectra in Figure S9c), we observe that the weakly H-bonded interfacial water signal first increases and then decreases with a rising OH_ad_/CO_a_ ratio during cathodic polarization, before sharply increasing again during the anodic return to low overpotentials, where only minimal CO_ad_ remains. This nonmonotonic behavior closely correlates with the surface restructuring dynamics observed by EC-AFM, with interfacial disorder coinciding with both the initial and renewed nanocluster formation during anodic return (Figures 2a–d and S5). Notably, the most pronounced surface restructurings occur under mixed CO and OH coadsorption, underscoring the crucial synergistic role of OH_ad_ in driving nanoscale Cu surface restructuring during CO reduction.

To validate the proposed Cu surface restructuring under the effect of OH_ad_ during COR, we performed grand canonical genetic algorithm (GCGA)^62^ global optimization of Cu(100) with varying OH and CO coverages across a range of relevant chemical potentials. The minima searches yielded a grand canonical ensemble representation of the system, containing 8097 unique surface phases with CO coverage up to 0.5 ML and OH up to 0.7 ML (Figure S10a). Figure 3a shows the color map of surface roughness, measured by the standard deviation of vertical Cu surface atom displacement as a function of OH and CO coverages. The most significant surface roughening occurs in the region corresponding to a mixed and intermediate coverage of CO and OH, similar to the case of Cu terraces under mixed H and CO coverage.^63^ However, under relevant conditions, the global minimum configuration of the system is either OH-only or CO-only. If the system were to follow the thermodynamics and fully equilibrate, it would never access the mixed coverage states (Figure S10b), contradicting the experimentally observed mixed coverage state up to −0.5 V_RHE_ (Figure 2j,k). Moreover, OH adsorption is thermodynamically unfavorable at more reducing potentials,^64,65^ yet experiments show persistent OH coverage even at very negative potentials (Figure 2j,k). Such behaviors indicate strong kinetic trapping effects in our system (full discussion in Supporting Information Note S1) so that it can retain OH on the surface and be stranded in the metastable mixed coverage regime within the experimental time scale. This kinetically trapped state is essential for observed surface restructuring. It should be noted that our findings highlight the importance of having access to experimental operando structural and chemical information on such catalysts since they reveal that standard thermodynamic models cannot be used to address such complex dynamic systems.

**Figure 3:**
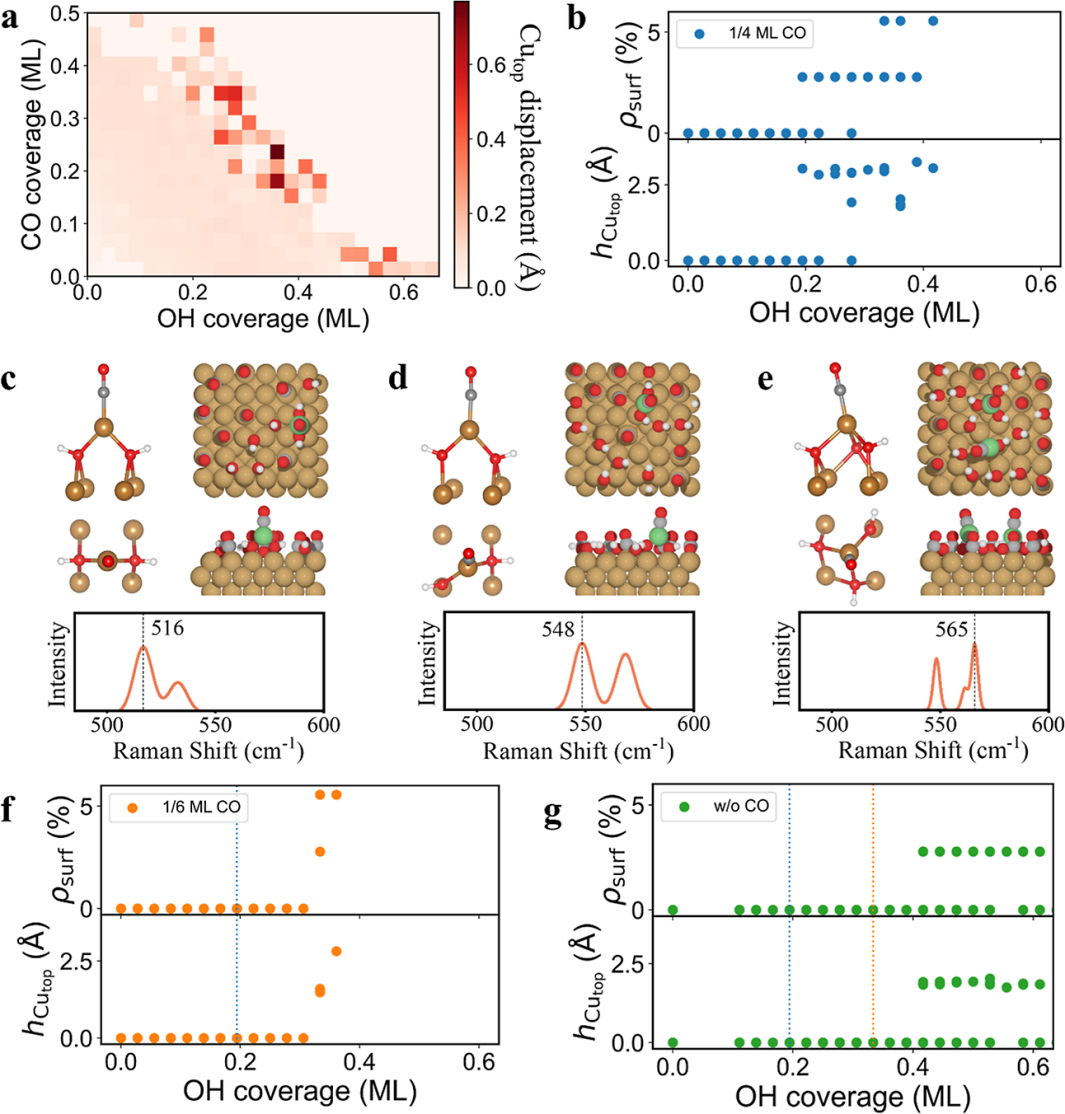
Theoretical insights into Cu surface restructuring under mixed CO and OH coverage. (a) Standard deviation of the top-layer Cu height, a metric of their vertical displacement as a function of OH and CO coverages. (b) Density (ρ_surf_) and relative height (*h*Cu_top_) of adatoms in accessible metastable surface phases under varying OH coverage and 1/4 ML CO coverage. (c–e) Representative phase structures with calculated Raman spectrum correspondingly under different extents of restructuring, OH coverages (c) 0.19 ML OH, OH/CO = 0.78; (d) 0.31 ML OH, OH/CO = 1.22; (e) 0.42 ML OH, OH/CO = 1.67. (f) Density (ρ_surf_) and relative height (*h*Cu_top_) of adatoms in accessible metastable surface phases under varying OH coverage and 1/6 ML CO coverage. (g) Density and height of adatoms in accessible metastable surface phases with varying OH coverage without any CO coverage.

Additional constrained GCGA samplings at 1/4 ML CO were performed to obtain a more extensive ensemble of mixed coverage states (Supporting Information Note S2), containing 11,412 unique surface phases. Within the ensemble, we could probe the effect of increasing OH coverage on surface roughening at a constant CO coverage, as a controlled variable approach (Figure 3b). Despite the lack of a quantitative relationship, it is known that OH coverage increases during the cathodic scan, and the key OH coverage values in our simulation can provide insight into the potential onset of surface phenomena. The key geometric characteristics for the low-energy local minima of each coverage state, as a function of the OH coverage, are shown in Figure 3b. At 1/4 ML CO coverage, the surface Cu atoms stay unreconstructed up to about 0.2 ML OH coverage, beyond which adatoms begin to form (at ∼2.78% surface density). The formed adatoms are elevated from the pristine surface Cu atoms by up to 2.8 Å. At a higher OH coverage of about 0.4 ML, multiple adatoms start to form within the sampling box, leading to an even higher surface density of adatoms. This is in agreement with the experimental finding of Cu terrace roughening at −0.15 V_RHE_, and more significant nanocluster formation with a larger amount of OH_ad_ is observed at −0.35 V_RHE_ (Figures 1 and S3). It can be seen that while thermodynamically not expected, OH could still be present on the surface at high cathodic potentials by being in a kinetically trapped state with Cu.

Figure 3c–e shows the structures of the representative surface phases with adatoms, further visualizing the chemical state of the formed low-coordinated Cu sites under different OH/CO coverages. All adatoms share a similar coordination environment, comprising one atop CO and several OH ligands attached to the surface, with the general formula of CuCO­(OH)_*n*_. At 0.19 ML OH coverage (Figure 3c), the adatom adopts the ligand shell CuCO­(OH)_2_, with both of the OH ligands binding to surface Cu atoms in the bridge mode. The remaining surface Cu atoms retain their pristine configuration, while the adatom is closer to the surface due to partial Cu–Cu interactions from undercoordination.

Raman calculations for this structure predict a prominent peak at ∼516 cm^–1^, corresponding to the bending mode of OH_ad_ on the Cu adatom (Figure S11a). At 0.31 ML OH coverage (Figure 3d), the adatom coordination remains CuCO­(OH)_2_, however, one OH ligand switches to an atop binding mode, due to increased OH coverage crowding the surface. The rest of the surface Cu atoms undergo a p4g-like restructuring locally near the vacancy. The dominant OH_ad_ vibrational mode shifts to ∼548 cm^–1^, reflecting the bending mode of bridged-bonded OH_ad_ on resconstructed surface atoms (Figure S11b). At 0.42 ML OH coverage (Figure 3e), multiple adatoms can form within the sampling box and bind to an additional OH, adopting the configuration CuCO­(OH)_3_, which is further elevated from the surface. The OH_ad_ bending modes further shift to ∼561 cm^–1^ on the extensively reconstructed surface. This aligns with our experimental SHINERS spectra, where the OH_ad_ bending mode evolves from ∼490 cm^–1^ to 530 cm^–1^ as surface restructuring progresses. Note that in aqueous solution, OH_ad_ forms H-bonds with interfacial H_2_O, which can cause experimental vibration frequencies to red-shift relative to the calculated values.

Notably, the formed Cu adatoms are intrinsically metastable and may be too transient to detect with the temporal resolution of EC-AFM, but they are expected to be the initiators of surface roughening. Over longer time scales, the EC-AFM-observable species, which dominate the surface and exhibit reduced mobility, are supposed to have stronger Cu–Cu interaction, and therefore sit closer to the surface with fewer OH or CO ligands, and a lower height (up to monolayer thickness), in line with the experimentally measured corrugation. Interestingly, a subensemble of 9817 unique surface phases, sampled under similar conditions but constrained to a CO coverage of 1/6 ML, shows that adatoms can still form on the surface at lower CO coverage (Figure 3f). The minimal OH coverage required to form adatoms (termed the “onset coverage”) is 0.33 ML, which is higher than in the case of the 1/4 ML CO coverage.

To gain deeper insight into the formation of adatoms and the stabilizing role of CO, a subensemble comprising 2009 unique surface phases with OH-only coverage was constructed. Figure 3g shows that adatoms can still form even without CO coverage, albeit with a significantly higher onset OH coverage (0.42 ML) as compared to cases where also CO is present. This observation aligns with the experimental findings, which indicate a stronger stabilization effect of CO on OH-induced adatoms (Figure 2). The structure of OH-only induced adatoms also differs from those with CO coverage (Figure S12a). The formed adatom, identified as Cu­(OH)_2_, features two OH ligands that connect the Cu adatom to the surface Cu in an atop binding mode, which is distinct from the bridge mode preferred by CO-coordinated adatoms. The Cu­(OH)_2_ adatom, where OH is found to be on the atop site and can break off the surface more easily, is less elevated (2.2 Å) compared to the case of CuCO­(OH)_*n*_, due to the absence of a strong atop ligand CO. The atop-site configuration of Cu­(OH)_2_ exposes the adatom to the electrolyte, making it more prone to dissolution or aggregation. The smaller elevation also indicates the retention of the Cu–Cu interactions between the adatom and surface Cu and the weaker cationic character of the adatom without atop CO. This is evidenced by the Bader charge of +0.60 and +0.74 |e| for Cu in Cu­(OH)_2_ and CuCO­(OH)_2_, respectively (Table S1).

We also analyzed the OH-only surface phase of 0.42 ML coverage (Figure S12a) and computed the reaction profile for atop CO adsorption on the adatom (Figure S12b–e). The results show that the adatom is highly cationic and its bonding with OH is ionic. The adatom induced by OH alone is less cationic and less elevated. With atop CO, back-donation makes Cu more positively charged and binds OH more strongly. Without atop CO, the interaction between the adatom and surface Cu is found to be partially metallic and partially mediated via OH; while with atop CO, no Cu–Cu interactions are found, and the adatoms and surface Cu are linked only via OH. A detailed analysis and description of the chemical bonding are provided in Supporting Information Note S3. The theoretical results align with our EC-AFM observations, which reveal differences in the stability of reconstructed Cu sites at more negative potentials, depending on the presence of CO in the headspace (Figure 2). Note that without OH_ad_, CO cannot lift adatoms out of the surface: no such Cu–CO structures are found within our thermodynamic ensembles that contain only CO, or within the kinetically accessible ensembles of mixed CO and OH coverages. This is because CO cannot weaken and insert into surface Cu–Cu bonds per se, unlike OH. Taken together, these results suggest that, while surface OH_ad_ alone can induce adatom formation, CO significantly enhances both their formation and stability. A higher CO supply reduces the onset OH coverage required for Cu restructuring, stabilizes the reconstructed Cu sites, and promotes further evolution.

Our findings reveal a synergistic mechanism by which OH_ad_ and CO_ad_ drive atomic-scale copper surface restructuring (Figure 4). While current works do assume the presence of OH_ad_ at thermodynamically expected regions,^34,66^ we actually show that OH_ad_ species become trapped with reconstructed Cu species at mild cathodic potentials, forming CuCO­(OH)_2_ adatom complexes that evolve into (sub)­monolayer nanoclusters (Figure 4a,b). Potential-controlled exposure, together with in situ gas-exchange experiments, rules out the reduction of preformed oxides as the origin of surface restructuring. Co-adsorption of *in situ*-generated OH with CO at cathodic potentials emerges as a key factor in driving the formation of mobile adatoms and unlocking surface restructuring. The subsequent fate of these adatoms—whether they form stable clusters or undergo dissolution—is then modulated by the local chemical environment (e.g., pH, potential). Supported by theoretical calculations, we propose that adsorbed OH withdraws electron density from Cu, partially oxidizing the Cu atom to Cu^+^ while remaining adsorbed. This partial oxidation weakens Cu–Cu bonds and lowers the energetic barrier for surface atom migration or dissolution. At highly cathodic potentials, where the CO_ad_/OH_ad_ coverage ratio is low, low-coordinated Cu sites tend to coalesce into larger Cu islands, accompanied by the formation of extended surface vacancies (Figure 4c). The stability of these nanoclusters is largely governed by the CO partial pressure and the CO_ad_/OH_ad_ coverage ratio. Notably, OH_ad_ remains irreversibly bound to atomically roughened Cu surface,^15,38^ as evidenced by its persistent signal even 1 h after CO depletion under Ar saturation (Figure S13). These results suggest that COR in alkaline media primarily occurs on roughened Cu surfaces with stabilized Cu–OH_ad_ species, which may indicate the presence of locally oxidized sites and Cu^+^ species under reducing potentials. The irreversble trapping of OH_ad_, coupled with the loss of low-coordinated nanoclusters, may also account for the poisoning effects observed at excessively high OH_ad_ coverages.^26,67^

**Figure 4:**
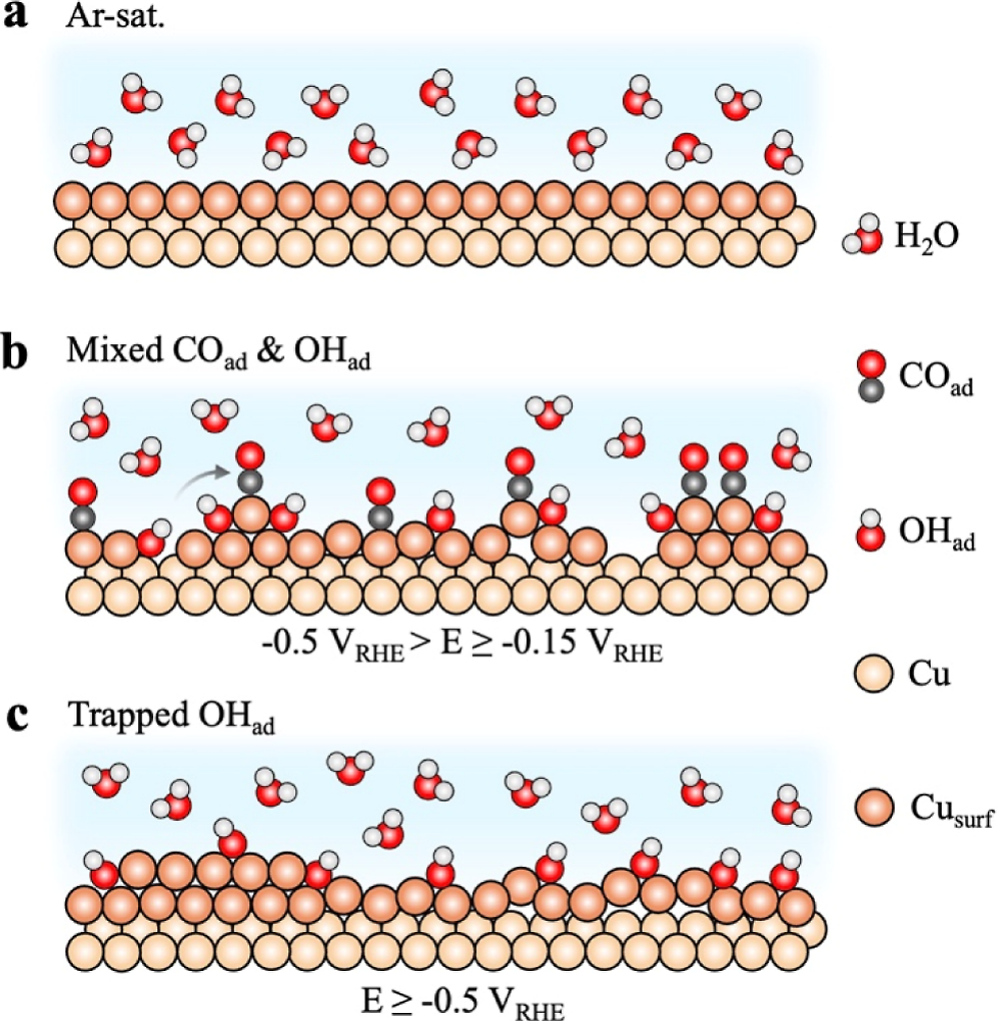
Schematic of the role of surface hydroxyls in atomic-scale Cu restructuring. (a) Atomically smooth Cu(100) surface with ordered interfacial H_2_O structure in Ar-sat. 0.1 M KOH. (b) Restructuring into low-coordinated Cu atoms/nanoclusters under mixed CO_ad_ and OH_ad_ coverage in CO-sat. 0.1 M KOH at mild cathodic potentials, accompanied by disruption of the interfacial H-bond network. (c) Ripening of Cu nanoclusters at highly cathodic potentials with a high OH_ad_/CO_ad_ coverage ratio, alongside OH_ad_ trapping on the roughened surface.

## Conclusions

We uncovered that surface hydroxyls (OH_ad_), ubiquitous in aqueous electrolytes, play a decisive role in driving Cu surface restructuring during CO electroreduction. Through a combination of in situ EC-AFM, SHINERS, and DFT calculations with grand canonical sampling, we showed that mixed OH and CO coadsorption at cathodic potentials promotes the formation of metastable Cu species, such as low-coordinated adatoms/nanoclusters, leading to atomic-scale roughening of the initial, atomically smooth terraces. This restructuring is accompanied by a more disordered, weakly H-bonded interfacial water network. At more negative potentials, large vacancies and/or islands emerge via aggregation, dissolution, and redeposition, which are suppressed under constant CO partial pressure. The potential-dependent stability, density, and size of (sub)­monolayer Cu nanoclusters are primarily governed by the OH_ad_/CO_ad_ coverage ratio, which is crucial for bridging the gap between UHV-based mechanistic studies and realistic electrochemical environments. Moreover, in contrast to the prevailing view that OH_ad_ only exists in thermodynamically favorable regions,^34,66^ we demonstrated that it can be kinetically retained: coadsorbing with CO at cathodic potentials to unlock atomic-scale surface restructuring, and remaining trapped on roughened, partially oxidized Cu surfaces even after CO depletion under highly cathodic potentials.

This work highlights the necessity for in situ structural and chemical information, as standard thermodynamic models alone cannot capture the complexity of dynamic electrocatalyst systems. Particularly, the observation of kinetically trapped Cu–OH_ad_ species underscores the importance of surface kinetics effects in rationalizing electrode structures and catalytic behavior. The low-coordinated Cu nanoclusters potentially provide preferred binding sites for critical reaction intermediates toward C_2_^+^ products and are inherently relevant for rationalizing COR activity and selectivity.^19,34^ By identifying the local electrochemical environment - specifically the kinetic balance of CO_ad_ and OH_ad_ coadsorption as key factors for both the formation and stability of these sites, we provide a critical foundation for understanding electrode restructuring at relevant potentials. These insights inform strategies for achieving operando control over catalytic selectivity and stability through precise tuning of electrolyte composition, pH, and reactant partial pressures.

## Supplementary Material



## Data Availability

Computational inputs and data sets are accessible at 10.5281/zenodo.17550260.

## Notes

Detailed experimental methods, electrode preparation, characterizations (EC-AFM imaging, electrochemical Raman measurements, etc.), computational methods (set-up, DFT calculations, grand canonical global optimization), and additional EC-AFM, EC-Raman figures, computational results and supplementary notes (PDF)
