# Supplementary material for: Role of Surface Hydroxyls in Atomic-Scale Copper Restructuring during CO Electroreduction
Source: J Am Chem Soc. 2025 Nov 24;147(49):45178–88. doi: 10.1021/jacs.5c14516 (PMC12703730; doi:10.1021/jacs.5c14516)
Supplement: Supplementary file 1 [file ja5c14516_si_001.pdf]

Supporting Information for

The role of surface hydroxyls in atomic-scale copper restructuring during CO electroreduction

*Jie Wei[a,b], Zisheng Zhang[c,e], Winston Gee[c], Yu Wei[c], Yawei Zhou[a,b], Matias Herran[b], Philippe Sautet[c,d], Anastassia N. Alexandrova[c], Beatriz Roldan Cuenya[b], Christopher S. Kley[a,b]**

[a] Helmholtz Young Investigator Group Nanoscale Operando CO_2 Photo-Electrocatalysis, Helmholtz-Zentrum Berlin für Materialien und Energie GmbH, 14109 Berlin, Germany

[b] Department of Interface Science, Fritz Haber Institute of the Max Planck Society, 14195 Berlin, Germany

[c] Department of Chemistry and Biochemistry, University of California, Los Angeles, CA 90095, USA

[d] Department of Chemical and Biomolecular Engineering, University of California, Los Angeles, CA 90095, USA

[e] SUNCAT Center for Interface Science and Catalysis, Department of Chemical Engineering, Stanford University, Stanford, California 94305, United States

*Corresponding author. Email: christopher.kley@helmholtz-berlin.de

Methods

Chemicals and Materials. Cesium hydroxide monohydrate (99.95% trace metals basis), potassium hydroxide (99.99% trace metals basis), lithium hydroxide monohydrate (99.995% trace metals basis), potassium bicarbonate (99.95% trace metals basis), potassium sulfate (99.99% trace metals basis), sulfuric acid (96%), were purchased from Sigma-Aldrich. All chemicals were used, as received, without further purification. Argon (99.999%) and nitrogen (99.999%) were procured from Air Liquide. Carbon monoxide (99.97%) was purchased from Linde Gas Singapore Pte Ltd. Deionized water (18.2 MΩ·cm, Barnstead Type 1) was used for all the studies. Shell-isolated nanoparticles (SHINs) were prepared using previously-reported protocols.[1] Cu(100) single crystal of high purity (6N) was purchased from MaTecK GmbH and constitute the working electrode (WE) after preparation before each experiment. Ring-shaped platinum sheet (99.996% purity, Asylum Research) and silver wires (99.99% purity, Asylum Research) were used as counter (CE) and pseudo-reference electrodes (RE). The pseudo-reference electrode was calibrated against a standard RHE (miniHydroflex, Gaskatel) before each experiment and verified by cyclic voltammetry after the experiments. All potentials were converted to a scale vs. RHE. No iR compensation was applied, given the low uncompensated resistance ($R_u \approx 10\ \Omega$) e and currents (< 100 µA) in the most relevant potential window. The exposed geometric WE surface area amounts to 0.55 cm^{-2}, and the CE surface area is about twice as large.

Electrode Preparation. The Cu(100) single crystal used in this study was electropolished in 65% ortho-phosphoric acid (85% H_3PO_4, PanReac, AppliChem) at potentials between 2.5 and 3.0 V vs. a Ti wire counter electrode. At least three polishing steps of ten seconds each were applied before every experiment. After each electropolishing treatment, the crystal was rinsed with ultrapure water with the last polishing step followed by rinsing the crystals with diluted acid (cal. 0.1M

H_2SO_4). After rinsing the crystal was dry in nitrogen gas (5N) flow and assembled in the electrochemical cells. The surface structures of the as-prepared single-crystal electrode was checked using cyclic voltammetry (CV) in Ar-purged electrolytes in the EC-AFM cell without the immersed AFM sensor.

EC-AFM imaging. The as-prepared samples were mounted fast in an open three-electrode electrochemical cell (PEEK, FFKM) and then covered with 0.1 M H_2SO_4 prepared from ultrapure sulfuric acid (Merck). After connection with an SP-200 potentiostat (BioLogic) equipped with an ultra-low current module, a potential in the range of -1.15 to -0.8 V vs. Ag was applied and finally the electrolyte was exchanged under potential control to the electrolytes to be studied. EC-AFM images in the electrolytes, were obtained in amplitude modulation (AM-AFM) mode also known as intermittent contact mode. The commercial AFM (CypherES, Asylum Research / Oxford Instruments) is a sample scanning system equipped with photo-thermal excitation of the cantilever oscillation and optical detection (405 nm, 850 nm) as well as a closed sample compartment to control the atmosphere surrounding the sample and electrolyte[2]. The CO gas inlet was regulated by a precision flowmeter, and the outlet was connected to a dedicated exhaust line to safely vent the gas outside the laboratory. A calibrated CO gas monitor with an audible alarm was placed nearby to provide continuous atmospheric monitoring. The *in situ* exchange of electrolytes was achieved through the perfusion tubes inserted on the cantilever holder, which are extended close to the sample surface to ensure an effective exchange of local electrolytes. High-frequency cantilevers (ARROW UHF Au, NanoWorld AG) were used throughout this study after cleaning with a gentle argon plasma treatment before imaging. Spring constants of the used cantilevers were (8 ± 5) N/m and resonance frequencies were in the range of (650 ± 110) kHz in electrolytes. The used oscillation amplitude set points fall in the range of 0.1-5 nm in electrolytes. The data were

analyzed using the Gwyddion[3] software.

Electrochemical Raman measurements. The as-prepared Cu single-crystal electrodes were assembled in a home-built spectro-electrochemical cell made of Teflon and controlled by a Biologic SP240 potentiostat for electrochemical Raman measurements. Shell-isolated nanoparticles (SHINs) were drop-casted onto the Cu electrode surface and dried in argon gas (5N) flow. After immersing the surface with electrolytes, a linear sweeping from OCP to -0.15 V vs. RHE was immediately applied followed by the chronoamperometry protocol staying at -0.15 V vs. RHE. This procedure leads to high-quality surfaces with large and clean terraces, in consistency with the surfaces used for EC-AFM studies.

Electrochemical Raman measurements were performed with a Raman spectrometer (Renishaw, InVia Reflex) coupled with an optical microscope (Leica Microsystems, DM2500M) together with a motorized stage for sample tracking (Renishaw, MS300 encoded stage). Calibration of the system was carried out by using a Si(100) wafer (520.5 cm^{-1}). A HeNe laser (Renishaw, RL633, $\lambda = 633$ nm, Pmax = 17 mW, grating 1800 lines mm^{-1}), was used as an excitation source. The backscattered light was Rayleigh-filtered and directed to a CCD detector (Renishaw, Centrus). For the *in situ* electrochemical measurements, the excitation source (1% power) was focused on the surface of the sample, and Raman scattering signals were collected with a water immersion objective (Leica microsystems, 63x, numerical aperture of 0.9). The objective was protected from the electrolyte by a Teflon (FEP) film (Goodfellow, film thickness of 0.0125 mm), which was wrapped around the objective. All shell-isolated nanoparticle-enhanced Raman spectra (SHINERS) were acquired from an average of three repeated spectra with a collection time of 10 seconds each. The presented spectra were checked both at different spots and at extended times to ensure the consistency of the results.

Theoretical model Set-up. The Cu(100) surface is modeled by a 4-layer 6×6 supercell of Cu(100) termination with a cell dimension of 15.336 Å×15.336 Å (constructed with experimental lattice parameter from ref [4]). The bottom two layers of the slab are constrained as bulk regions, and everything else is allowed to relax as the interface region. A vacuum slab of 15 Å thickness is added in the Z direction to avoid spurious interactions between periodic images.

DFT calculations. The local optimizations and energy evaluation are performed with the RPBE functional[5] and PBE_PAW pseudopotentials[6] using the VASP program (version 5.4.4).[7-10] The convergence criteria for electronic and force minimization are set to 10-5 eV and 0.05 eV/Å during the global optimization and 10^{-6} eV and 0.01 eV/Å for the final refinement. Due to the relatively large system and sampling size, only the Γ k-point is sampled in the reciprocal space of the Brillouin zone throughout, and the cutoff energy for the kinetic energy of the plane waves was 400 eV. Grand canonical DFT calculations are performed on the low-energy local minima of every coverage state. The potential-dependent electronic grand canonical free energy of the surface (Ω_{el}) is obtained by the surface charging approach[11] and following the same procedures and parameters as in ref [12-14]. The transition states (TS) are located using climbing image nudged elastic band (CI-NEB) method[15] with image dependent pair potential (IDPP) interpolation[16]. Each TS geometry has been confirmed to have only one imaginary mode. All electronic structure analyses are performed based on converged charge density or wavefunction. The Bader charges are calculated using the Bader Charge Analysis program[17]. The QTAIM analysis is performed using the critic2 program using the Wigner-Seitz method with a subdivision level of 2[18].

Grand Canonical Global Optimization. To sample the chemical space of both Cu restructuring and adsorbate (CO and OH) coverage/configuration, we performed global optimization using the grand canonical genetic algorithm (GCGA) as implemented in our open-source GOCIA python

package (https://github.com/zishengz/gocia),[19] which now supports mixed and polyatomic adsorbates. To be specific, the system is treated as a grand canonical ensemble of H and CO adsorbates, and the search target is to minimize the coverage-dependent grand canonical free energy Ω_{ads}:

$$\Omega_{\mathrm{ads}} = U - TS - \sum \mu_i N_i \approx E^{\mathrm{slab}-n\mathrm{H}} - E^{\mathrm{slab}} - n_{\mathrm{CO}} \cdot \mu_{\mathrm{CO}}(p_{\mathrm{CO}}, T) - n_{\mathrm{OH}} \cdot \mu_{\mathrm{OH}}(\mathrm{pH}, U, T)$$

Where the $E^{\mathrm{slab}-n\mathrm{H}}$ and E^{slab} are electronic energies of the adsorbate-covered and the bare Cu(100) slab. The vibrational contributions to free energy by the slab atoms are neglected considering their small contribution and high computational cost[20]. The chemical potential of OH (μ_{OH}) is calculated from the free energy of water and the chemical potential of H, μ_{H} (pH, U, T). The pH and U (in SHE scale) dependent terms are calculated using the computational hydrogen electrode model[21]. The electronic energy terms are substituted with electronic free energies (Ω_{el} from GCDFT calculations) in the final ensemble. The ZPE and thermal contribution terms of adsorbates are obtained from frequency calculations and evaluated at 298.15 K.

The unconstrained GCGA searches use the settings for polyatomic adsorbates, with multiple sets of chemical potentials in the experimentally relevant range of electrochemical potential and CO partial pressure. The constrained GCGA is achieved by an outer iteration approach: the locally optimized structure will undergo additional addition/removal operations until it reaches the target adsorbate coverages, and then the new structure will be reoptimized until the coverage converges to the target.

Raman Calculation. We performed Raman calculations for the three structures with varying OH coverages shown in Figure 3. Local optimizations were carried out using the RPBE functional and PBE_PAW pseudopotentials as implemented in VASP (version 5.4.4). The convergence thresholds

for the electronic self-consistency and ionic relaxation were set to 1×10^{-5} eV and 0.05 eV Å^{-1}, respectively. A plane-wave kinetic-energy cutoff of 400 eV and a 2 × 2 × 1 Monkhorst–Pack k-point mesh was employed. Following structural optimization, frequency calculations were performed by displacing all surface adsorbed OH groups along the x, y, and z directions with uniform magnitude, while keeping all other atoms fixed. This approach restricts the vibrational analysis to the adsorbates of interest. For each vibrational mode, structures with positive and negative displacements were generated, and VASP calculations were used to evaluate the dielectric tensor for each displaced configuration. Off-resonant Raman activities were computed within the Placzek approximation by finite-difference differentiation of the macroscopic static dielectric tensor with respect to the normal coordinate of each vibrational mode. For a given mode iii with normalized eigenvector $e^{(i)}$ and frequency v_i, only the surface adsorbed OH groups were displaced along $\pm\Delta$ in each Cartesian direction, while all other atoms were kept fixed. The displaced geometries were generated as:

$$R^{(\pm)} = R_0 \pm \frac{\Delta}{\|e^{(i)}\|} e^{(i)}$$

For each displacement, a self-consistent VASP calculation was performed to obtain the macroscopic static dielectric tensor $\epsilon^{(\pm)}$. The Raman tensor R was then evaluated via central finite difference:

$$R_{mn}^{(i)} = \frac{\epsilon_{mn}^{(+)} - \epsilon_{mn}^{(-)}}{2\Delta} \times \|e^{(i)}\| \times \frac{V}{4\pi}$$

where m, n $\in \{x, y, z\}$ V is the cell volume, and the factor $\frac{V}{4\pi}$ converts the dielectric response to polarizability units.

From R_{mn}, the isotropic polarizability α and the anisotropic polarizability β^2 were computed as:

$$\alpha = \frac{R_{xx} + R_{yy} + R_{zz}}{3}$$

$$\beta^2 = \frac{1}{2}\left[\left(R_{xx} - R_{yy}\right)^2 + (R_{xx} - R_{zz})^2 + \left(R_{yy} - R_{zz}\right)^2 + 6\left(R_{xy}^2 + R_{xz}^2 + R_{yz}^2\right)\right]$$

Finally, the Raman activity S_i for mode i was obtained from the Placzek invariant:

$$S_i = 45\alpha^2 + 7\beta^2$$

The Raman spectrum was generated by applying Gaussian broadening to S_i over all computed modes.

Additional figures, notes, and tables

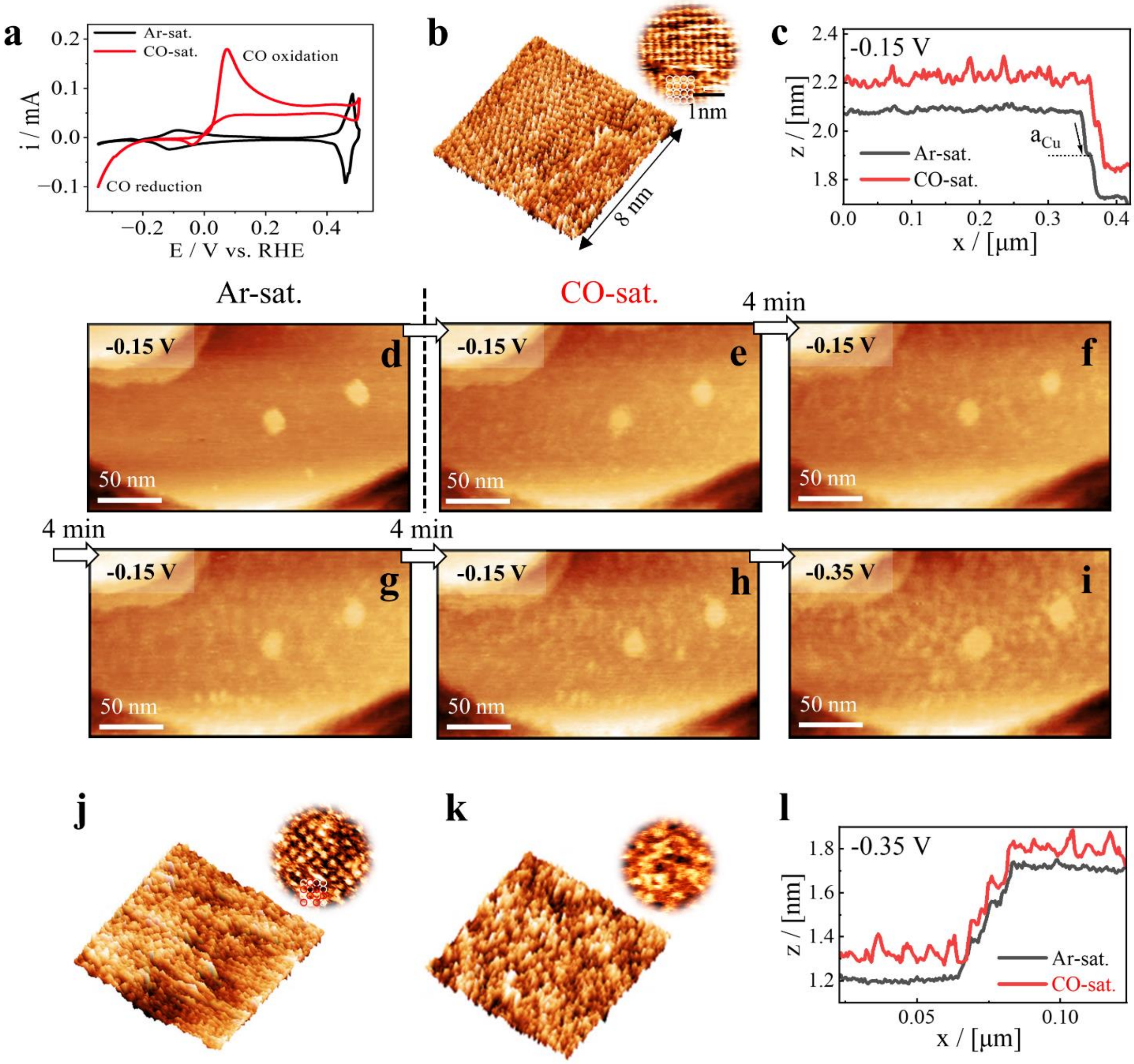

Figure S1. Atomic-scale images of Cu(100) surface reconstruction upon CO adsorption in 0.1 M KOH. (a) Cyclic voltammograms of Cu(100) electrode in Ar-sat. vs. CO-sat. 0.1 M KOH within the EC-AFM cell. (b) Atomic-resolution EC-AFM images recorded on the as-prepared Cu(100) surface at -0.15 V_{RHE} in Ar-sat. 0.1 M KOH. (c) Line profiles taken at the locations indicated by black horizontal lines in Figure 1b. The height data was leveled by mean plane subtraction on each terrace for better illustration. The arrow marks an atomic step. (d-i) Enlarged EC-AFM images taken from the white boxes marked in Figure 1b, demonstrating in detail the roughening process of the smooth terrace when CO was introduced in the solution and more pronounced formation of nano-structures with extended time. (j-k) Atomic-resolution EC-AFM images recorded in CO-sat. 0.1 M KOH with the surface showing firstly a coexistence of Cu-(1 x 1) and CO-c(2 x 2) structures at -0.15 V_{RHE} (j), and after more than 20 min the nano-structuring on the surface (k). (l) Line profiles taken at the locations indicated by horizontal lines in Figure 1c in main text.

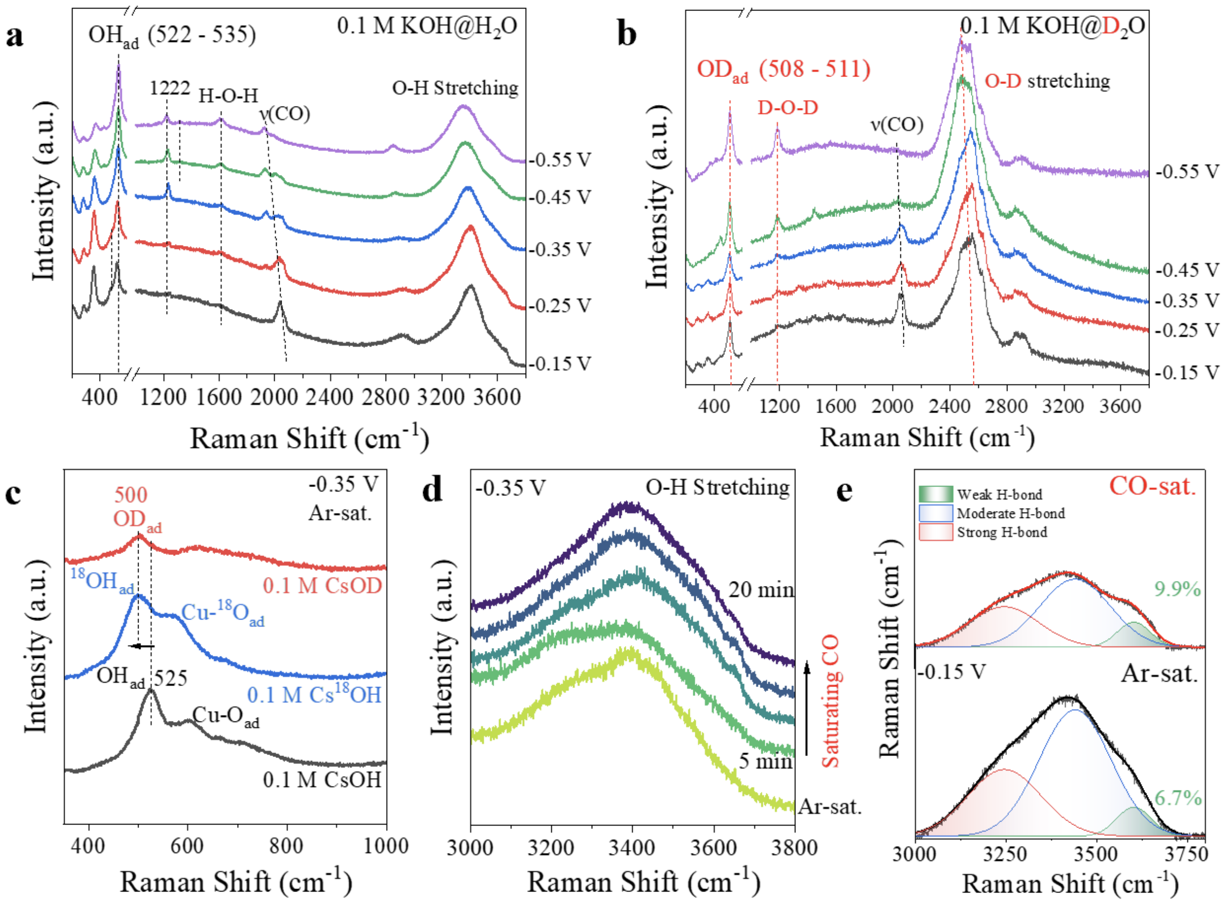

Figure S2. Normalized *in situ* (isotope labelling) SHINERS spectra recorded on the Cu(100) surface at different applied potentials in Ar-sat. and CO-sat. 0.1 M MOH solutions. (a-b) Comparison of potential dependent *in situ* SHINERS spectra in CO-sat. KOH (a) and KOD (b) solutions. (c) Normalized in situ SHINERS spectra recorded on the Cu(100) surface at -0.35 V_{RHE} in Ar-sat. 0.1 M CsOH/CsOD/CsO^{18}H solutions. (d) Evolution of O-H stretching mode after saturating the previous Ar-sat. 0.1 M KOH with CO at -0.35 V_{RHE}. (e) Comparison of the H-bonds among surface H_2O between Ar-sat. and CO-sat. 0.1 M KOH at -0.15 V_{RHE}.

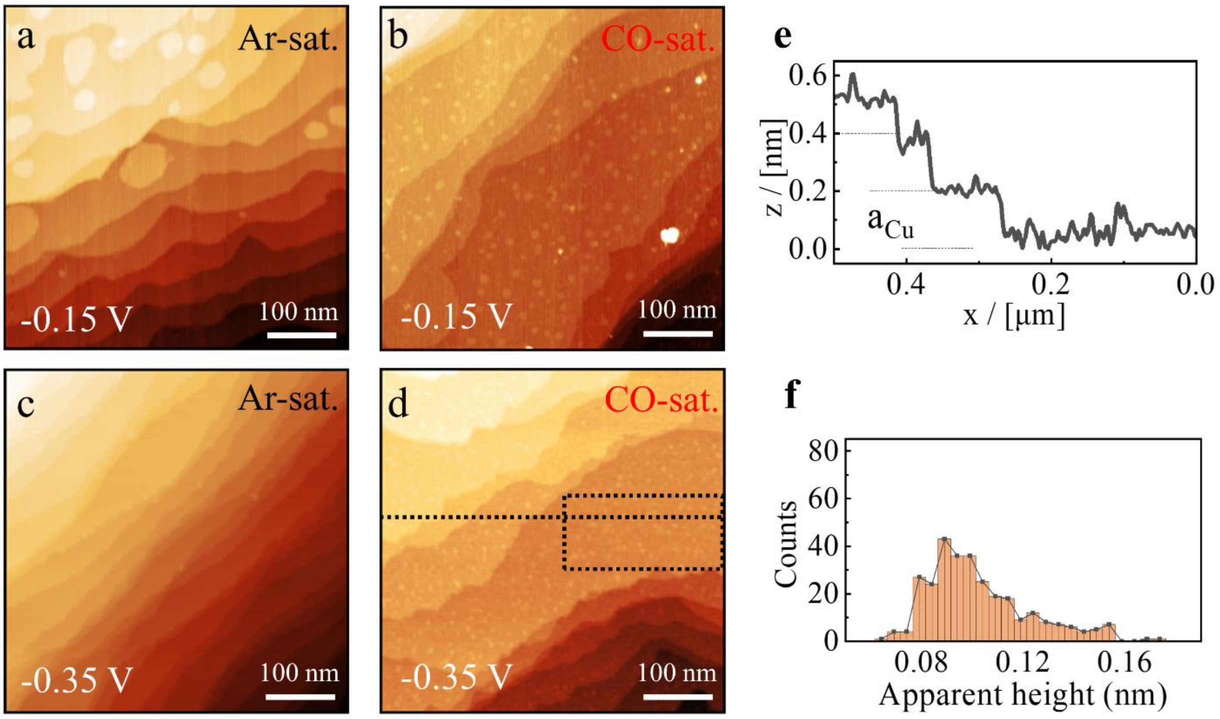

Figure S3. ***In situ*** **Cu(100) surface reconstruction in CO-sat. 0.1 M KOH. (a-d)** EC-AFM images of Cu(100) in Ar-sat. 0.1M KOH (a, c) and CO-sat. 0.1M KOH (b, d), at -0.15 V_{RHE} (a-b) and -0.35 V_{RHE} (c-d), showing a high population of nanocluster on the terrace in CO-sat. 0.1 M KOH. **(e)** Line profiles taken at the locations indicated by horizontal lines in (d). **(f)** The apparent height distribution of nanoclusters in the dashed box area of (d).

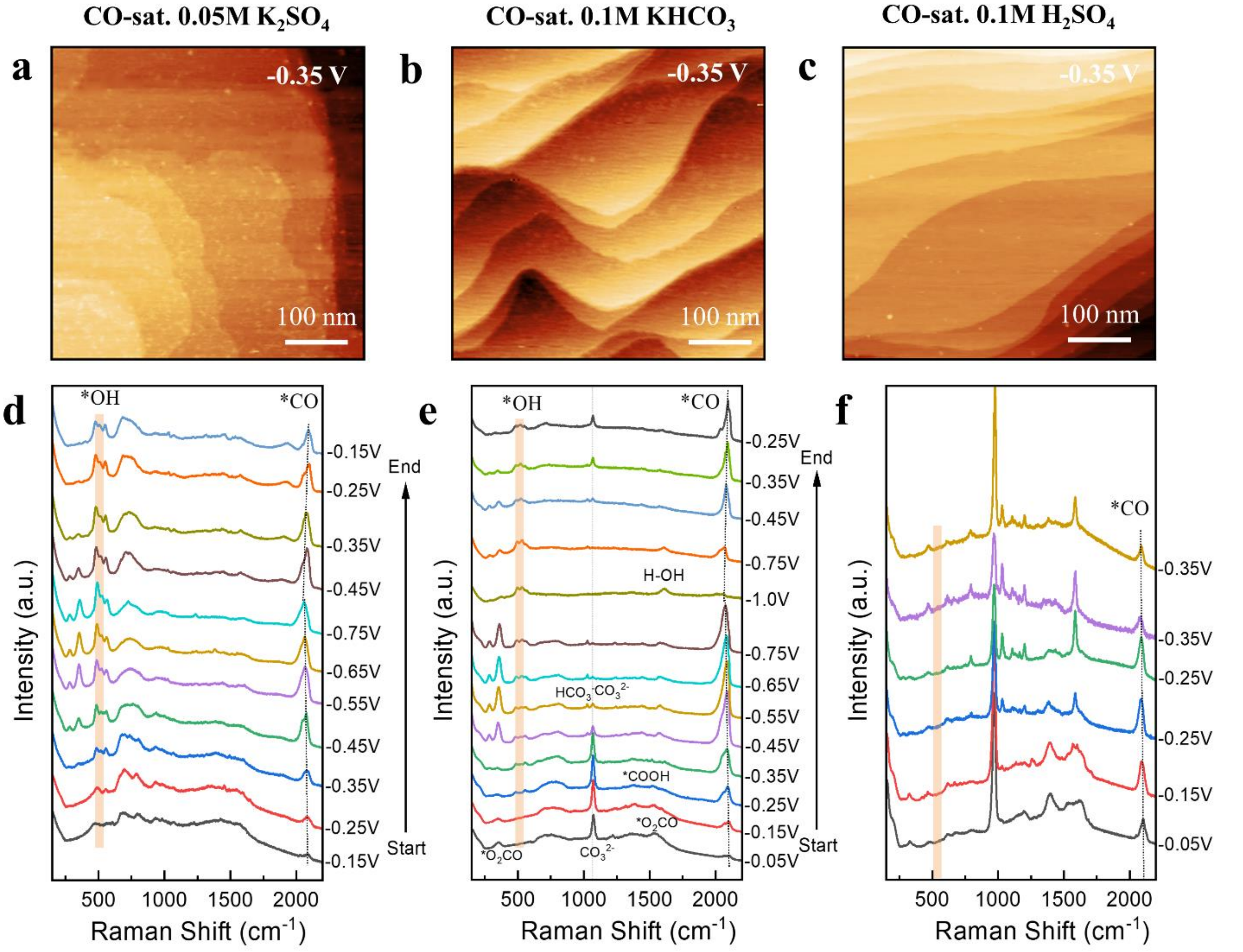

Figure S4. ***In situ*** **EC-AFM images and chemical information of Cu(100) surface in CO-sat. electrolyte with different pHs.** EC-AFM image at -0.35 V_{RHE} **(a-c)** and normalized *in situ* SHINER spectra at different applied potentials **(d-f)** recorded on the fresh-prepared Cu(100) in CO-saturated (a,d) 0.05 M K_2SO_4 (pH ≈ 7), (b,e) 0.1 M $KHCO_3$ (pH ≈ 8.9) and (c, f) 0.1 M H_2SO_4 (pH ≈ 1). The orange band marked the band position where surface OH_{ad} is assigned.

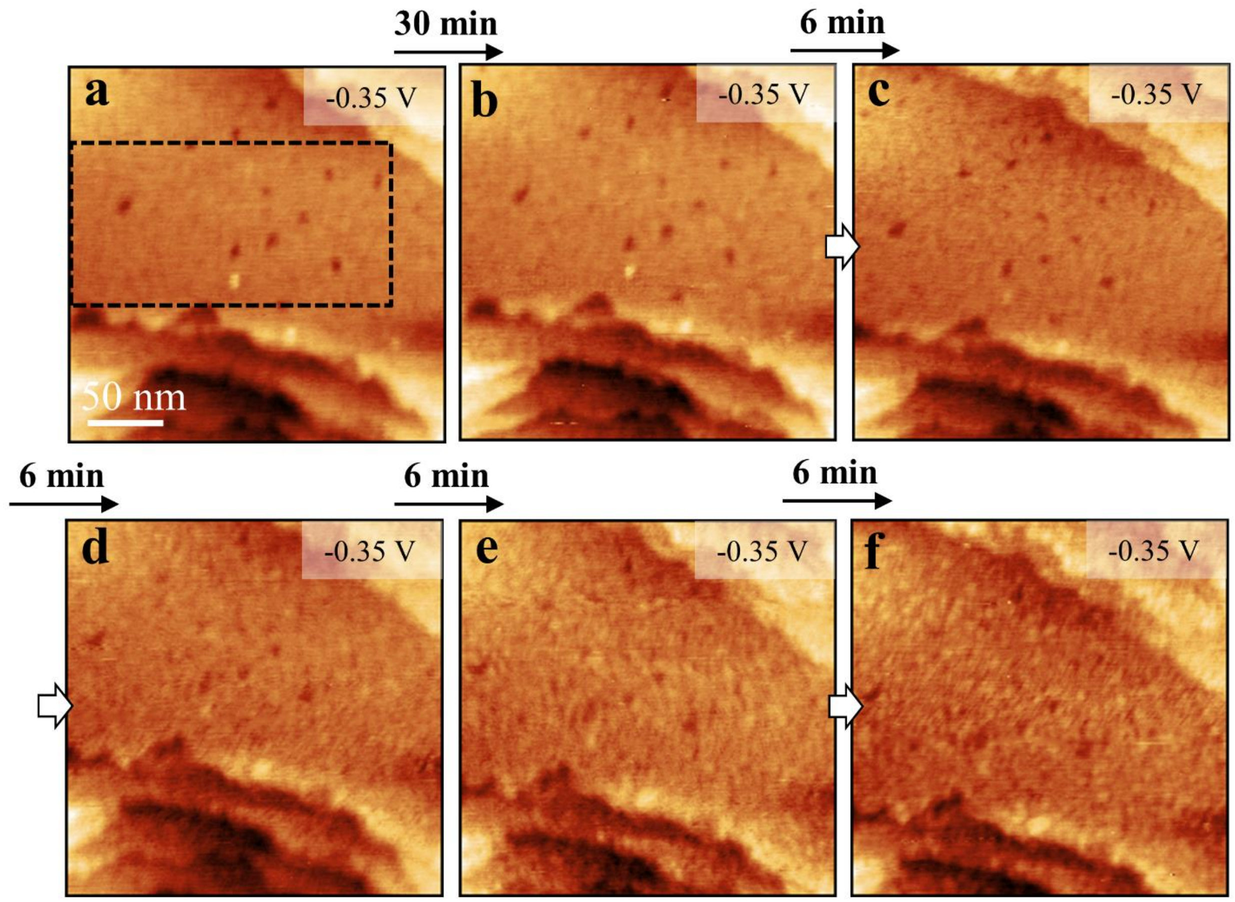

Figure S5. EC-AFM image sequences of as-prepared Cu(100) in the CO-sat. 0.1 M KOH after going back to mild cathodic potentials, showing again the nano-restructuring on the terrace with large vacancies. **(a)** Potential reverse to -0.35 V_{RHE}. **(b)** -0.35 V_{RHE} after 30 mins. **(c)** -0.35 V_{RHE} after 36 mins. **(d)** -0.35 V_{RHE} after 42 mins. **(e)** -0.35 V_{RHE} after 48 mins. **(f)** -0.35 V_{RHE} after 54 mins**.**

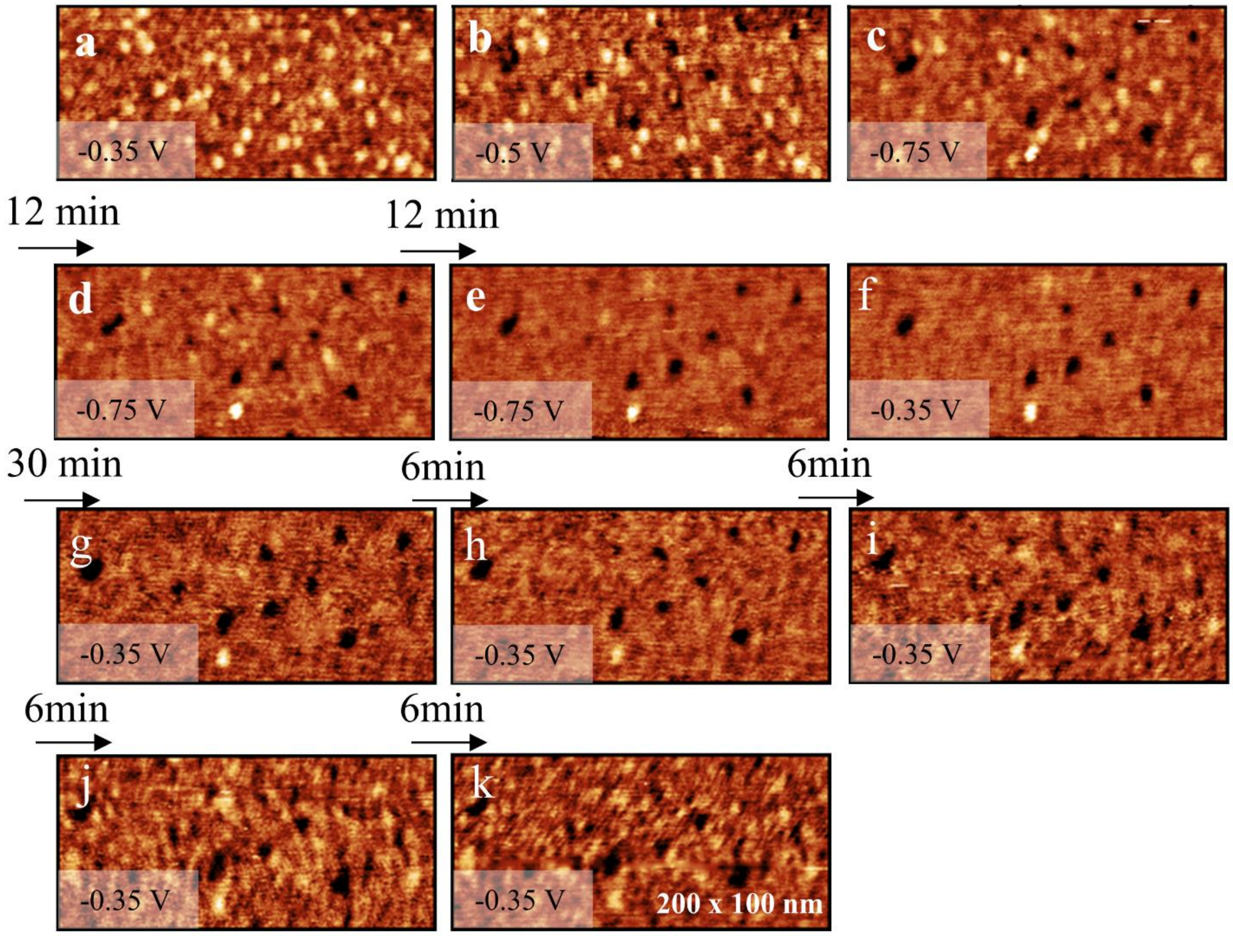

Figure S6. Enlarged EC-AFM images at different potentials and over time, showing the disappearance of Cu nanoclusters and formation of vacancies at highly cathodic potentials and again the nano-restructuring when going back to mild cathodic potentials. (a) -0.35 V_{RHE}. **(b)** -0.5 V_{RHE}. **(c)** -0.75 V_{RHE}. **(d)** -0.75 V_{RHE} after 12 mins. **(e)** -0.75 V_{RHE} after 24 mins. **(f)** potential reverse to -0.35 V_{RHE}. **(g)** -0.35 V_{RHE} after 30 mins. **(h)** -0.35 V_{RHE} after 36 mins. **(i)** -0.35 V_{RHE} after 42 mins. **(j)** -0.35 V_{RHE} after 48 mins. **(k)** -0.35 V_{RHE} after 54 mins**.**

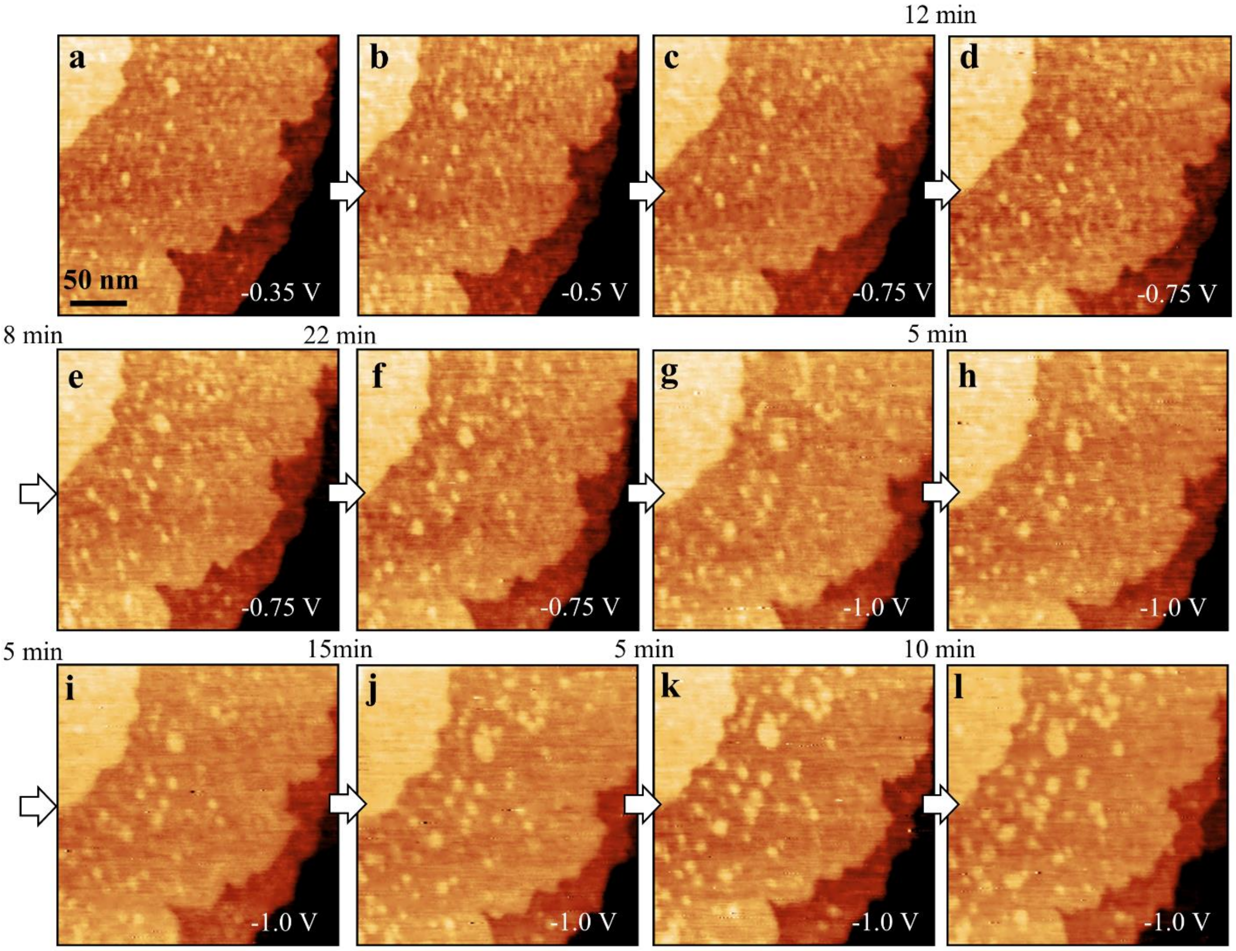

Figure S7. EC-AFM image sequences of as-prepared Cu(100) in the CO-sat. 0.1 M KOH under highly cathodic potentials, showing the *in situ* aggregation of nanoclusters to form large Cu islands on the terrace. **(a)** -0.35 V_{RHE}. **(b)** -0.5 V_{RHE}. **(c)** -0.75 V_{RHE}. **(d)** -0.75 V_{RHE} after 12 min. **(e)** -0.75 V_{RHE} after 20 min. **(f)** -0.75 V_{RHE} after 42 min. **(g)** -1.0 V_{RHE}. **(h)** -1.0 V_{RHE} after 5 min. **(i)** -1.0 V_{RHE} after 10 min. **(j)** -1.0 V_{RHE} after 25 min. **(k)** -1.0 V_{RHE} after 30 min. **(l)** -1.0 V_{RHE} after 40 min.

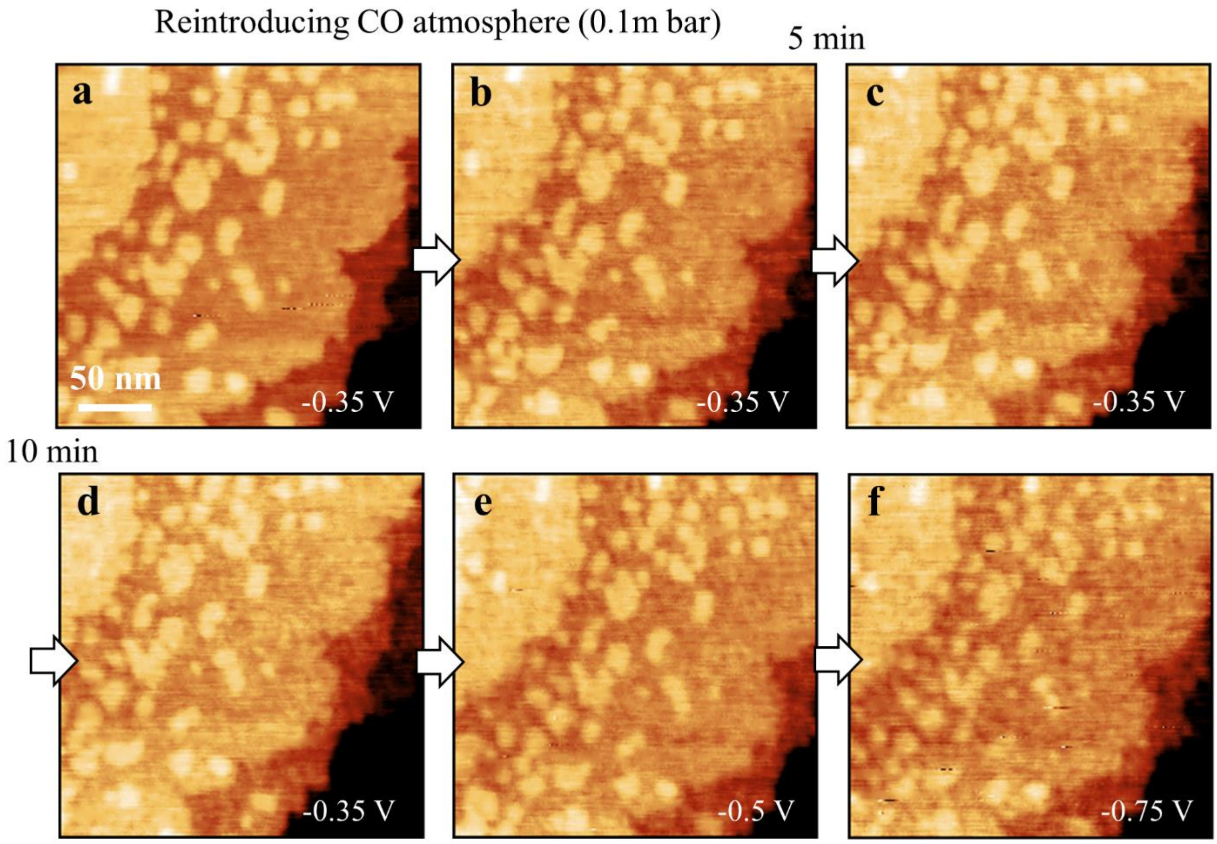

Figure S8. EC-AFM image sequences of Cu(100) in 0.1M KOH after introducing CO atmosphere through the headspace of AFM chamber. (a) Smooth Cu(100) terrace decorated with large Cu islands after long time COR. **(b-f)** Reconstruction of smooth terrace after CO adsorption over extended time and potentials. (b) -0.35 V_{RHE}. (c) -0.35 V_{RHE} after 5 min. (d) -0.35 V_{RHE} after 15 min. (e) -0.5 V_{RHE}. (f) -0.75 V_{RHE}.

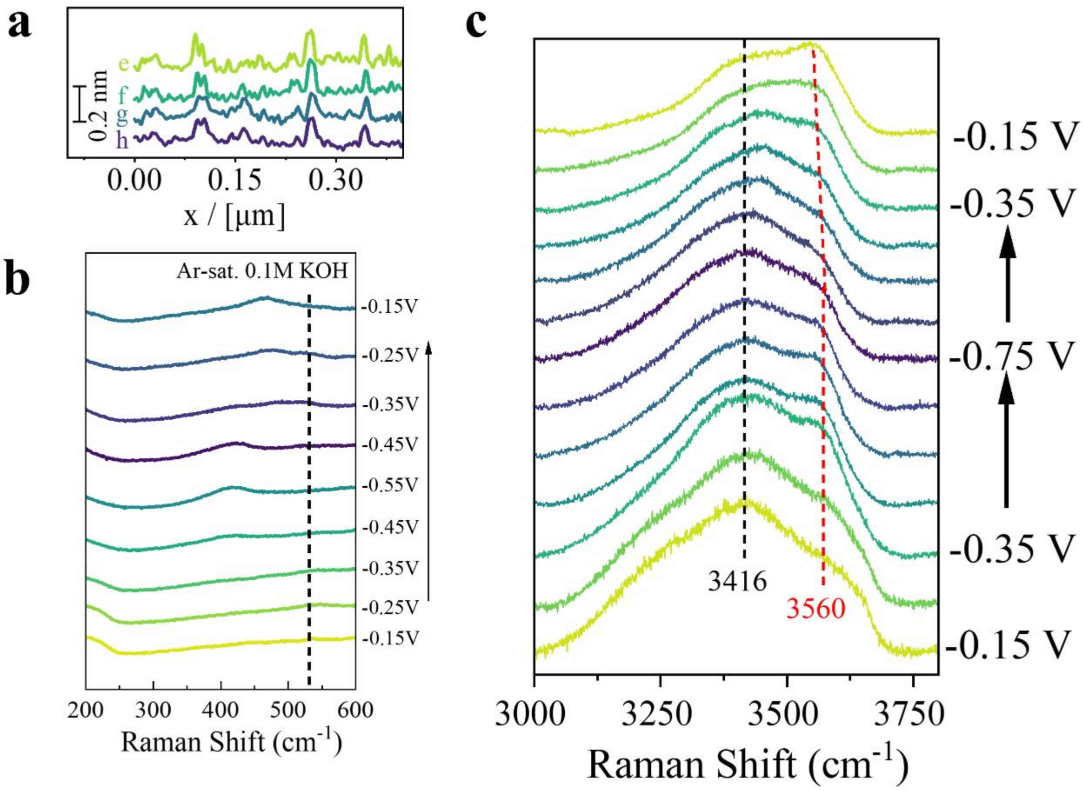

Figure S9. Molecular scale insights into Cu(100) in 0.1 M KOH with different gas saturation. **(a)** Height variations at the locations indicated by horizontal lines in (e-h**)** of Figure 2. Smooth Cu(100) terrace decorated with large Cu islands after long time COR. **(b)** Potential-dependent in situ SHINER spectra recorded on Cu(100) in Ar-sat. 0.1 M KOH. **(c)** Potential-dependent O–H stretching mode of interfacial water on Cu(100) surface in CO-sat. 0.1 M KOH.

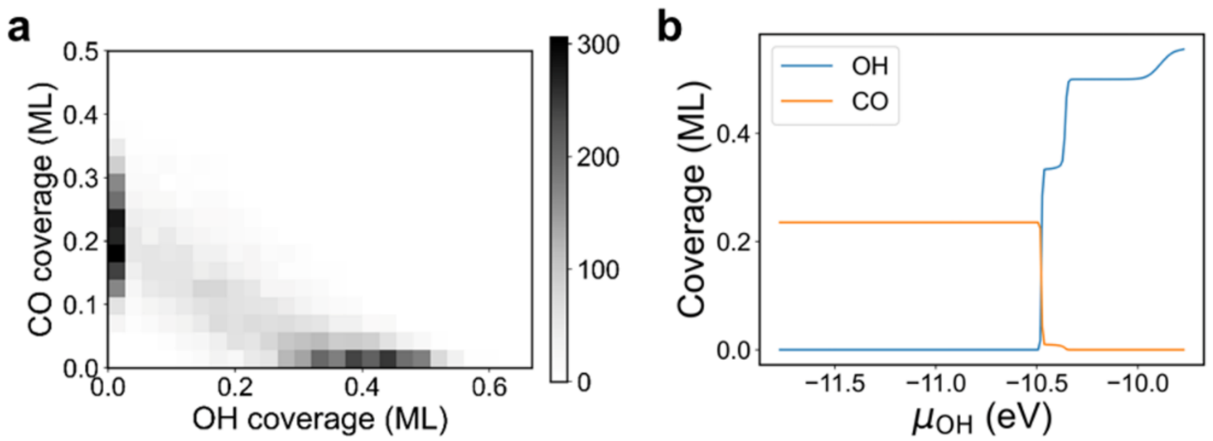

Figure S10. Results of the unconstrained GCGA searches. (a) Sampling frequency in the coverage regime. **(b)** CO and OH coverage as a function of the chemical potential of OH, in a range corresponding to electrochemical potentials from -1.5 to 0.5 V.

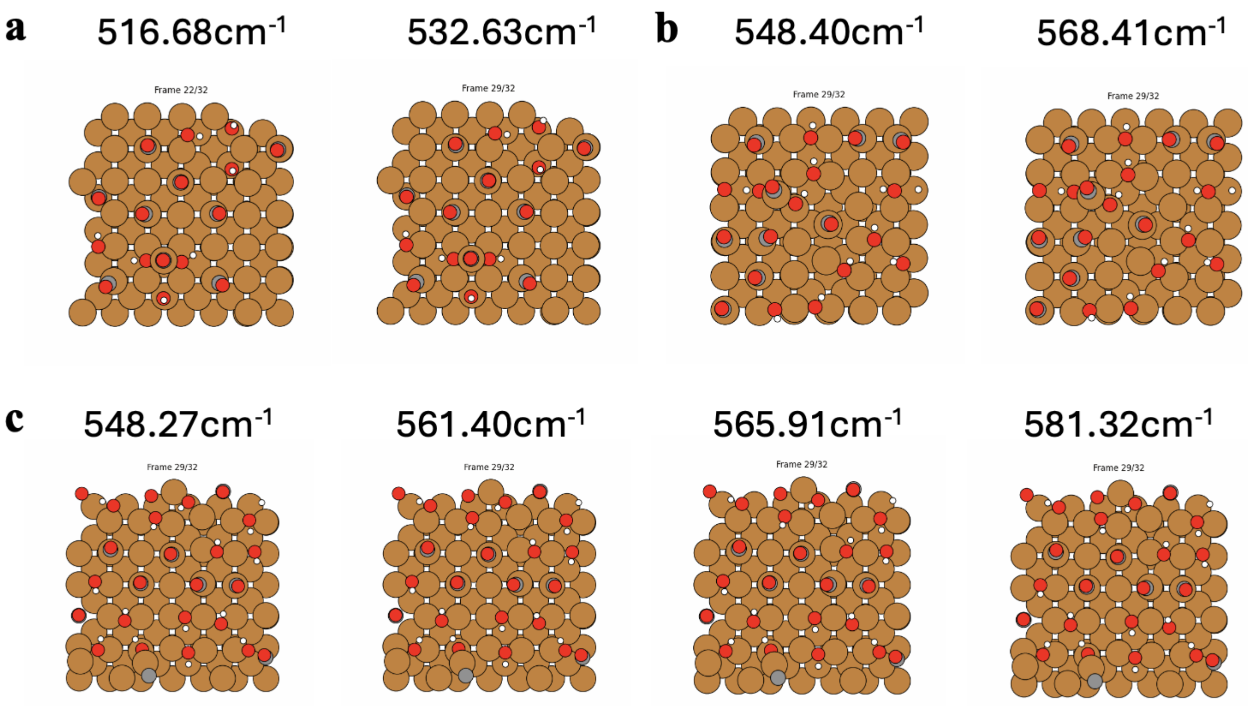

Figure S11. Calculated OH_{ad} bending mode on Cu surface with different extent of reconstruction and OH coverage. (a) One adatom; 0.19 ML OH and OH/CO = 0.78; **(b)** One adatom; 0.31 ML OH and OH/CO = 1.22; **(c)** Two adatoms; 0.42 ML OH and OH/CO = 1.67.

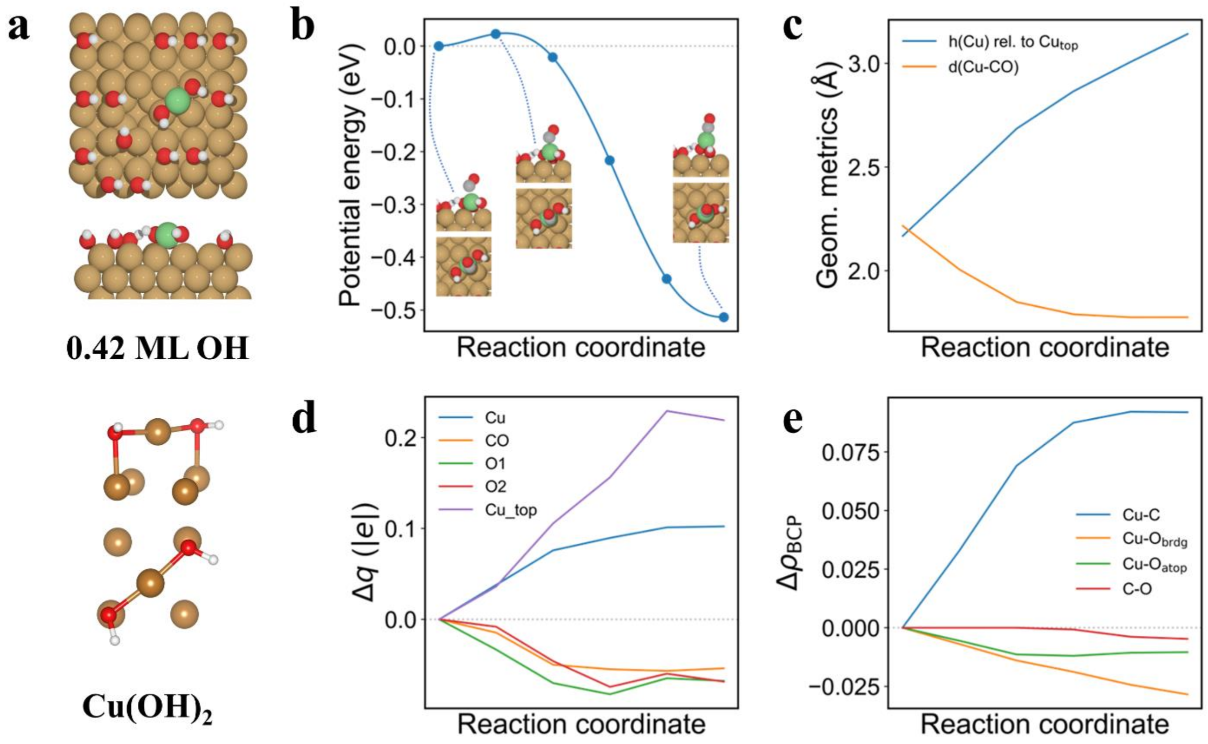

Figure S12. Chemical bonding analysis of the CO binding on $Cu(OH)_2$. (a) The structure of the onset surface phases with adatoms of Cu(100) restructuring under pure OH coverage. **(b)** The energy profile of CO binding on $Cu(OH)_2$, from physiosorbed to chemisorbed configurations. The structures of the initial, transition, and final states are shown in the inset. **(c)** The geometric change in the height of Cu adatoms relative to the top surface Cu and the Cu-CO bond length along the reaction coordinate. **(d)** The change in Bader charges on key atoms and motifs along the reaction coordinate. **(e)** The change in charge density at bonding critical points for notable bonds along the reaction coordinate.

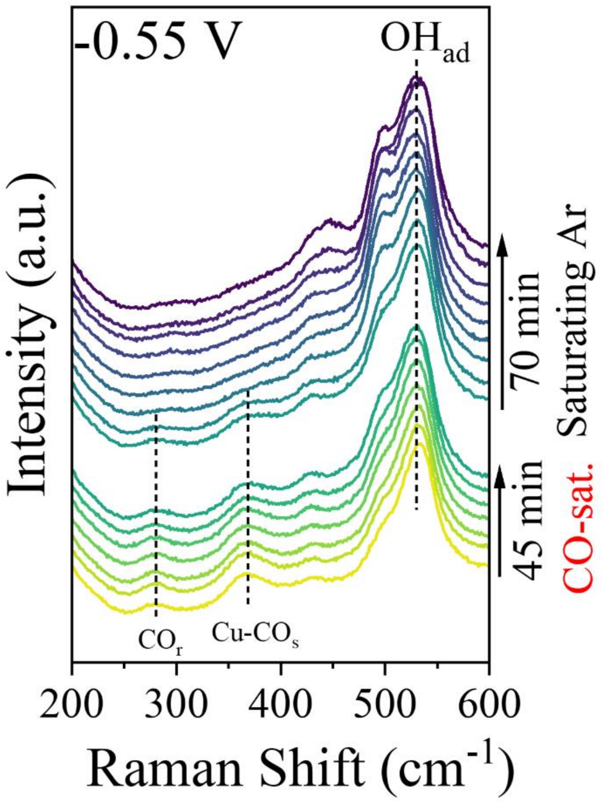

Figure S13. Time-dependent in situ SHINER spectra recorded on Cu(100) in CO-sat. 0.1 M KOH and after CO depletion.

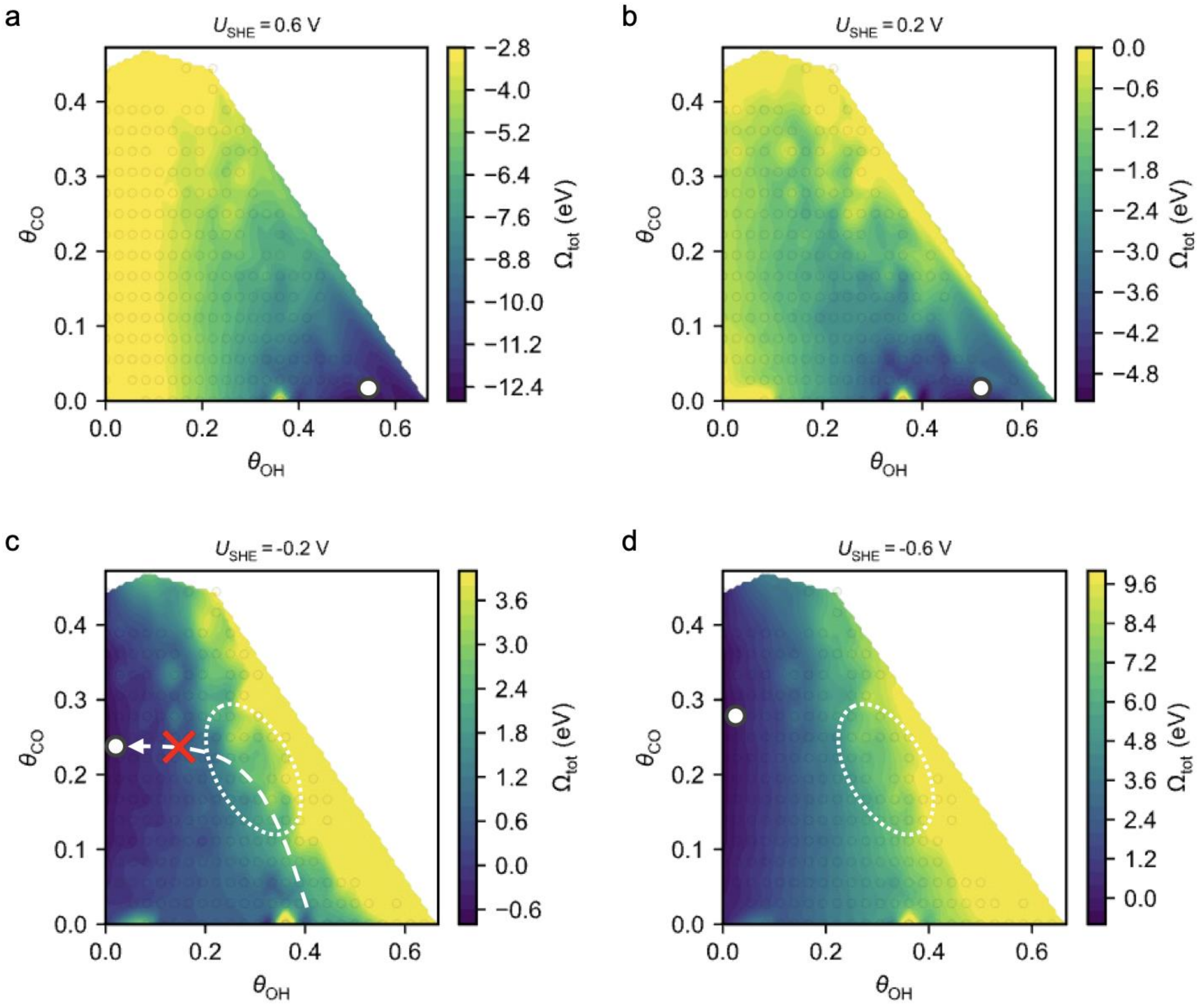

Figure S14. Total grand canonical free energy landscapes in the coverage space at various potentials. The potential is varied from oxidative to reductive, at (a) +0.6 V, (b) +0.2 V, (c) -0.2 V, (d) -0.6 V, in the SHE scale. The white marker marks the global minimum state in each plot. The white dashed curve illustrates the kinetically limited path from OH-only minimum to CO-only minimum. The white dotted circle marks the probably regime of kinetic trapping.

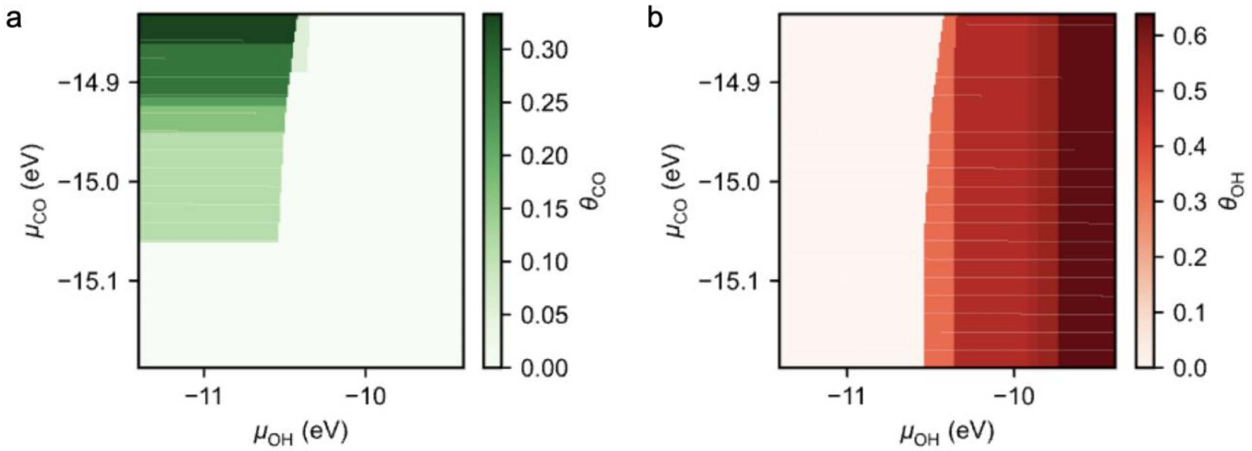

Figure S15. Thermodynamic surface phase diagrams of OH and CO coverages at varying chemical potentials of OH and CO. The range of μ_{CO} corresponds to from 10^{-6} to 1 atm, and the range of μ_{OH} corresponds to from -1 to +1 V_{SHE} at pH of 7.

	Cu	C in CO	O in CO	O in OH	H in OH
Cu(100)	0.00				
Cu(100) w/ *CO	0.15	0.89	-1.06		
Cu(100) w/ *OH	0.28			-1.18	0.59
$CuCO(OH)_2$	0.74	0.92	-1.02	-1.22	0.62
$Cu(OH)_2$	0.60			-1.15	0.65
$Cu(OH)_2$+CO	0.68	1.02	-1.10	-1.14	0.63
$Cu(OH)_2$*CO	0.79	0.92	-1.05	-1.21	0.63

Table S1: **The Bader charge on key atoms in notable surface phases.** "+" and "*" stand for physisorption and chemisorption, respectively.

Supporting Information Note S1. Discussion of the kinetic trapping and non-Boltzmann behaviors

The concept of "kinetic trapping" refers to the system staying in a thermodynamically metastable regime in catalytic time scales, i.e., the system is not ergodic and cannot equilibrate to the global thermodynamic minimum structure. The origin of such behavior lies in the grand canonical free energy landscape of the Cu surface under varying OH and CO coverages. In oxidative environments (**Figure S14a, b**), the system is the most stable in the OH-only coverage regime. In reducing conditions **(Figure S14c, d)**, the CO-only coverage regime becomes the most stable.

If the system were to be ergodic and always equilibrate to the global minimum, then the evolution of adsorbate coverage and surface phase would depend solely on the thermodynamics (**Figure S15**). In catalytically relevant conditions, would exist either as a pure *CO-covered pristine Cu surface or as a pure *OH-covered surface with less favorable surface roughening, with a “cliff” in between the two, and completely skipping all mixed coverage states. Moreover, under highly reducing potentials, thermodynamics predicts that the system should be in the CO-only coverage states were the Cu surface returns to the non-restructured pristine arrangement.

However, the experiments observe (i) OH coverage even in the reducing potential regime, and (ii) significant surface restructuring. Both behaviors contradict the thermodynamic equilibrium picture. Hence, we infer that the system suffers strong kinetic limitation on its way from OH-only minima to CO-only minima (dotted path in **Figure S14c**), and it is highly likely stranded in a mixed coverage regime which is thermodynamically metastable and with sufficient local minima to trap the system indefinitely (encircled region in **Figure S14c, d**).

In summary, the *CO + *OH coexisting regime – which drives favorable surface roughening – represents a metastable state in the free energy landscape. The persistence of this metastable configuration at thermodynamically unstable potentials is critical for unlocking nanoscale Cu surface restructuring.

Supporting Information Note S2. Additional GCGA sampling.

The ensemble shown in Figure S9 is considered to be under-sampled in the metastable mixed coverage regime due to the thermodynamics-only nature of the GC global optimization method, biasing the sampling towards the coverages of the global minima (either CO-only or OH-only). Therefore, we performed additional GCGA searches with the CO coverage constrained at 1/4 ML and OH coverage sampled, so as to sample more extensively the chemical space of Cu(100) under a mixed coverage. The additional searches yielded a sub-ensemble containing 11,412 unique surface phases.

Supporting Information Note S3. Reaction profile computation for atop CO adsorption on the adatom.

Figure S10a shows that the chemisorption of CO turns out to be highly exothermic (reaction energy: -0.51 eV) and almost barrierless (barrier: 0.02 eV), showing the kinetic and thermodynamic favorability of CO binding on adatom. In Figure S10b, we can see that the major geometric changes are: the formation of the Cu-CO bond; elevation of Cu from 2.2 to 3.1 Angstrom; and shift of one OH ligand's surface-linking mode from atop to bridge. The Bader charges of key atoms are shown in Figure S10c. The gain of positive charge on Cu adatom and negative charge on CO are roughly the same (about 0.1 |e|), suggesting Cu-to-CO electron transfer. This, combined with the weakening of the C-O bond in the CO binding process (Figure S9c), reveals the back-donation to be the main driving force in stabilizing the more elevated and cationic Cu species and in offsetting the breaking of Cu-Cu bonds between the adatom and the surface. As the Cu elevates and becomes more cationic, the OH ligands, in response, draw more electrons from the top surface Cu. This is coupled with the shifts of surface-linking mode from atop to bridge which maximizes interaction and charge transfer (Bader charge on O: -1.14 |e| in atop OH; -1.22 |e| in bridge OH). In the meantime, the top surface Cu atoms that are bonded to the OH ligands become more positively charged by about 0.2 |e| in total. Note that the seeming weakening of bonds between OH and surface Cu in Figure S10d is due to the fact that each bridge OH binds to two surface Cu (whereas atop OH can only bind to one). Hence, the overall anchoring of adatom is strengthened by both electrostatics and a doubled number of bonds. The above factors cause the adatom to be more stable and less prone to clustering in the presence of a CO-rich environment as compared to CO-deficient or CO-free ones.

References

1. Li, J. F.; Huang, Y. F.; Ding, Y.; Yang, Z. L.; Li, S. B.; et al. Shell-isolated nanoparticle-enhanced Raman spectroscopy. *Nature* **464**, 392-395 (2010).
2. Simon, G. H.; Kley, C. S.; Roldan Cuenya, B. Potential-Dependent Morphology of Copper Catalysts During CO_2 Electroreduction Revealed by In Situ Atomic Force Microscopy. *Angew. Chem., Int. Ed.* **60**, 2561-2568 (2021).
3. Nečas, D.; Klapetek, P. Gwyddion: an open-source software for SPM data analysis. *Open Physics* **10**, 181-188 (2012).
4. Janthon, P.; Luo, S.; Kozlov, S. M.; Viñes, F.; Limtrakul, J.; Truhlar, D. G.; Illas, F. Bulk Properties of Transition Metals: A Challenge for the Design of Universal Density Functionals. *J Chem Theory Comput* **10**, 3832-3839 (2014).
5. Hammer, B.; Hansen, L. B.; Nørskov, J. K. Improved adsorption energetics within density-functional theory using revised Perdew-Burke-Ernzerhof functionals. *Phys. Rev. B* **59**, 7413 (1999).
6. Kresse, G.; Joubert, D. From ultrasoft pseudopotentials to the projector augmented-wave method. *Phys. Rev. B* **59**, 1758 (1999).
7. Kresse, G.; Furthmüller, J. Efficiency of ab-initio total energy calculations for metals and semiconductors using a plane-wave basis set. *Comp Mater Sci* **6**, 15-50 (1996).
8. Kresse, G.; Furthmüller, J. Efficient iterative schemes for ab initio total-energy calculations using a plane-wave basis set. *Phys. Rev. B* **54**, 11169 (1996).
9. Kresse, G. Ab initio molecular dynamics for liquid metals. *J Non-Cryst Solids* **192**, 222-229 (1995).
10. Kresse, G.; Hafner, J. Ab initio molecular-dynamics simulation of the liquid-metal–amorphous-semiconductor transition in germanium. *Phys. Rev. B* **49**, 14251 (1994).
11. Steinmann, S. N.; Michel, C.; Schwiedernoch, R.; Sautet, P. Impacts of electrode potentials and solvents on the electroreduction of CO_2: a comparison of theoretical approaches. *Phys. Chem. Chem. Phys.* **17**, 13949-13963 (2015).
12. Zhang, Z.; Gee, W.; Sautet, P.; Alexandrova, A. N. H and CO Co-Induced Roughening of Cu Surface in CO_2 Electroreduction Conditions. *J. Am. Chem. Soc.* **146**, 16119-16127 (2024).
13. Zhang, Z.; Wei, Z.; Sautet, P.; Alexandrova, A. N. Hydrogen-induced restructuring of a Cu (100) electrode in electroreduction conditions. *J. Am. Chem. Soc.* **144**, 19284-19293 (2022).
14. Poths, P.; Li, G.; Masubuchi, T.; Morgan, H. W.; Zhang, Z.; Alexandrova, A. N.; Anderson, S. L. Got coke? Self-limiting poisoning makes an ultra stable and selective sub-nano cluster catalyst. *ACS Catal.* **13**, 1533-1544 (2023).
15. Henkelman, G.; Uberuaga, B. P.; Jónsson, H. A climbing image nudged elastic band method for finding saddle points and minimum energy paths. *J. Chem. Phys.* **113**, 9901-9904 (2000).
16. Smidstrup, S.; Pedersen, A.; Stokbro, K.; Jónsson, H. Improved initial guess for minimum energy path calculations. *J. Chem. Phys.* **140**, 214106 (2014).
17. Yu, M.; Trinkle, D. R. Accurate and efficient algorithm for Bader charge integration. *J. Chem. Phys.* **134**, 064111 (2011).
18. Otero-de-la-Roza, A.; Johnson, E. R.; Luaña, V. Critic2: A program for real-space analysis of quantum chemical interactions in solids. *Comp Phys Com* **185**, 1007-1018 (2014).
19. Zhang, Z.; Gee, W.; Lavroff, R. H.; Alexandrova, A. N. GOCIA: a grand canonical global optimizer for clusters, interfaces, and adsorbates. *Phys. Chem. Chem. Phys.* **27**, 696-706 (2025).
20. Sun, G.; Alexandrova, A. N.; Sautet, P. Structural rearrangements of subnanometer Cu oxide clusters govern catalytic oxidation. *ACS Catal.* **10**, 5309-5317 (2020).
21. Yang, T. T.; Patil, R. B.; McKone, J. R.; Saidi, W. A. Revisiting trends in the exchange current for hydrogen evolution. *Catal. Sci. Technol.* **11**, 6832-6838 (2021).
